# The 1994 North American Interagency Intercomparison of Ultraviolet Monitoring Spectroradiometers

**DOI:** 10.6028/jres.102.021

**Published:** 1997

**Authors:** Ambler Thompson, Edward A. Early, John DeLuisi, Patrick Disterhoft, David Wardle, James Kerr, John Rives, Yongchen Sun, Timothy Lucas, Tanya Mestechkina, Patrick Neale

**Affiliations:** National Institute of Standards and Technology, Gaithersburg, MD 20899-0001, USA; National Oceanic and Atmospheric Administration, 325 Broadway, Boulder, CO 80303, USA; Atmospheric Environment Service, Environment Canada, 4905 Dufferin Street, Toronto, ON M3H 5T4, Canada; Dept. Physics and Astronomy, University of Georgia, Athens, GA 30602, USA; Biospherical Instruments Inc., 5340 Riley Street, San Diego, CA 92110-2621, USA; Smithsonian Environmental Research Center, P.O. Box 28, Edgewater, MD 21037, USA

**Keywords:** environmental monitoring, intercomparison, solar ultraviolet, spectroradiometers

## Abstract

Concern over stratospheric ozone depletion has prompted several government agencies in North America to establish networks of spectroradiometers for monitoring solar ultraviolet irradiance at the surface of the Earth. To assess the ability of spectroradiometers to accurately measure solar ultraviolet irradiance, and to compare the results between instruments of different monitoring networks, the first North American Intercomparison of Ultraviolet Monitoring Spectroradiometers was held September 19–29, 1994 at Table Mountain outside Boulder, Colorado, USA. This Intercomparison was coordinated by the National Institute of Standards and Technology and the National Oceanic and Atmospheric Administration (NOAA). Participating agencies were the Environmental Protection Agency, National Science Foundation, Smithsonian Environmental Research Center, and Atmospheric Environment Service, Canada. Instruments were characterized for wavelength accuracy, bandwidth, stray-light rejection, and spectral irradiance responsivity, the latter with a NIST standard lamp calibrated to operate in the horizontal position. The spectral irradiance responsivity was determined once indoors and twice outdoors, and demonstrated that, while the responsivities changed upon moving the instruments, they were relatively stable when the instruments remained outdoors. Synchronized spectral scans of the solar irradiance were performed over several days. Using the spectral irradiance responsivities determined with the NIST standard lamp, and a simple convolution technique to account for the different bandwidths of the instruments, the measured solar irradiances agreed within 5 %.

## 1. Introduction

Concern over stratospheric ozone depletion has prompted several government agencies in North America to establish networks of spectroradiometers for monitoring solar ultraviolet irradiance at the surface of the Earth. Since each agency has its own programmatic objectives, different instruments are deployed in each network at different geographical locations. All the networks, however, share the same goals of determining the current solar ultraviolet irradiance climatology and detecting long-term changes in this irradiance [1].

Both of these goals require accurate measurements of the irradiance in SI units, especially for the detection of long-term trends. This is necessary for individual instruments, and when achieved by all the instruments within a network enables the detection of such changes over large geographical areas. This geographical coverage is extended still further, and the ability to detect trends in a consistent manner is improved, by comparing the results from different networks.

To assess the ability of spectroradiometers to accurately measure solar ultraviolet irradiance and to compare these results between instruments of different monitoring networks, the first North American Intercomparison of Ultraviolet Monitoring Spectroradiometers was held September 19–29, 1994, outside Boulder, Colorado. This Intercomparison was coordinated by the Optical Technology Division (formerly the Radiometric Physics Division) of the National Institute of Standards and Technology and the Surface Radiation Research Branch (SRRB) of the National Oceanic and Atmospheric Administration (NOAA). Spectroradiometers from monitoring networks administered by the following agencies participated: the Environmental Protection Agency (EPA), the National Science Foundation (NSF), the Smithsonian Environmental Research Center (SERC), and the Atmospheric Environment Service (AES) of Canada. A list of attendees is given in Appendix A.

During the Intercomparison, instrument parameters were characterized that affect the accurate measurement of solar ultraviolet irradiance and that did not require elaborate experimental techniques, namely wavelength accuracy, stray-light rejection, the slit-scattering function, and spectral irradiance responsivity. The last characterization both checked the absolute irradiance scales used by the networks and provided a common scale for the synchronized measurements of solar irradiance. These synchronized measurements were the most important aspect of the Intercomparison as they assess the present limits to which irradiances determined by different instruments can be compared. Other instruments determined the atmospheric conditions during the Intercomparison, which will be useful for correlating these conditions with the measured solar ultraviolet irradiance. A list of all the instruments present at the Intercomparison is given in Table 1.1.^1^ Note that all times given in this paper are in Universal Coordinated Time (UCT), which was 6 h ahead of Mountain Daylight Time, the local time.

Intercomparisons similar to the one described here have been performed previously by other countries, primarily those from Europe [2–8]. The planning of the North American Intercomparison benefited greatly from the results reported by these other Intercomparisons. Common to all were assessments of spectral irradiance responsivity and synchronized solar irradiance measurements by a variety of instruments. The North American Intercomparison, however, was limited to participants with instruments deployed in operating monitoring networks, and emphasized determination of the spectral irradiance responsivity of all the instruments several times with a common lamp standard, and using these results for the synchronized solar irradiance measurements.

## 2. Site Description

The site of the Intercomparison was Table Mountain, a plateau owned by the Federal government approximately 12.9 km north of Boulder, Colorado and 5.6 km east of the front range of the Rocky Mountains. This site was chosen because of its good view to the horizon, the presence of laboratory facilities, and the proximity of facility and staff support at both NIST and NOAA in Boulder.

For the synchronized measurements of solar irradiance, the spectroradiometers were located on individual concrete pads on the south side of the plateau at latitude 40.125° N, longitude 105.237° W, and elevation 1689 m. The pads were arranged in an east-west line and were 2.4 m square and 12.2 m between centers. The highest, and only major, obstruction to the horizon was a peak 5.6 km due west of the pads with an angle of inclination of 5.1°. Temporary trailers approximately 30 m south of the pads housed the data acquisition and control computers and equipment for the spectroradiometers. The plateau sloped downward south of the pads, so the tops of the trailers were below the elevation of the pads. A test facility platform approximately 30 m west of the west-most pad is NOAA’s SRRB site. At the Intercomparison, pyranometers, pyrgeometers, pyrheliometers, and shadowband radiometers were located on the platform. A meteorological tower recording the temperature, relative humidity, atmospheric pressure, and wind speed and direction at the site was located approximately 90 m northwest of the pads. Finally, a concrete building immediately to the southwest of the platform was used for servicing the instruments, holding meetings, and performing indoor characterizations. A dome at the western end of the building was covered with a black cloth to eliminate reflections from it to the instruments.

## 3. Instrument Descriptions

Five instruments were used for the Intercomparison. Two Brewer Spectrophotometers, Model MKII, serial numbers 009 and 113, were operated by the participants from AES Canada. The instrument from the EPA network was also a Brewer Spectrophotometer, Model MKIV, serial number 109, and was operated by participants from the University of Georgia, who manage the EPA network. The NSF instrument was a Biospherical Instruments SUV-100 Ultraviolet Spectroradiometer, serial number B-007, operated by participants from that company, who also administer the NSF network. Participants from SERC operated a Smithsonian SR-18 Ultraviolet Scanning Radiometer, serial number UC. For the remainder of this paper, these instruments will be designated AES-1, AES-2, EPA, NSF, and SERC, respectively. With the exception of the SERC instrument, the spectroradiometers operate by scanning a specified wavelength range, in which the monochromator is set at a certain wavelength, signal is accumulated, the wavelength of the monochromator advances by a specified interval, and the process is repeated. Table 3.1 lists the characteristics of each instrument, and detailed descriptions are given below, including the algorithms used to calculate the spectral irradiance from the measurement. Note that these algorithms are supplied by the participants, and no attempt has been made to critically evaluate their suitability.

### 3.1 Brewer Spectrophotometer

The Brewer Spectrophotometer measures total solar ultraviolet irradiance from 290 nm to 325 nm (Model MKII) or from 286.5 nm to 363 nm (Model MKIV) and total column O_3_ and SO_2_ from both direct sun and zenith sky measurements at specific ultraviolet wavelengths. The Model MKIV also determines total column NO_2_. The instrument is housed in a weatherproof container constructed of two pieces: a base to which all optical and electronic assemblies are anchored and a removable cover. The container is vented to the atmosphere through a canister containing desiccant. The instrument is mounted on an azimuth tracker, an all-weather positioning pedestal with a chassis containing a large stepper motor, drive electronics, and gearing. The tracker in turn is mounted on the vertical axis of a tripod foundation.

**Fig. 3.1:**
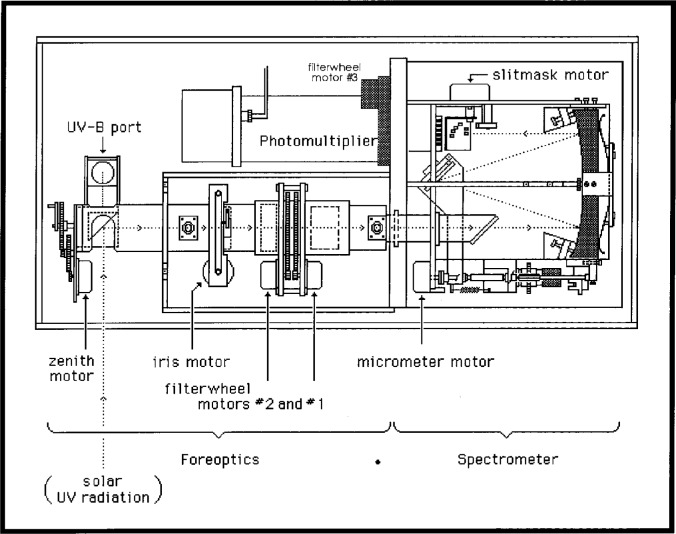
Plan view of the major optical assemblies and the optical path of a Sci-Tec Brewer Spectrophotometer Model MKIV.

A plan view of the major optical assemblies and the optical path is shown in Fig. 3.1. Light is directed through the foreoptics by a right-angle zenith prism which rotates to select one of several sources. Direct light from the sun or zenith sky is collected through an inclined quartz window when the prism is at zenith angles from 0° to 90°. Light from calibration lamps, one a 20 W quartz-halogen lamp that provides a well-regulated light source to monitor the relative spectral response of the instrument and the other a Hg emission lamp for wavelength calibration, is collected when the prism is at a zenith angle of 180°. A lens between the quartz-halogen lamp and the prism collimates the light from this lamp along the optical axis, and the Hg lamp is underneath the quartz-halogen lamp. A thin, 3.2 cm diameter flat disk of Teflon mounted under a 5 cm diameter quartz dome on top of the instrument cover collects the total irradiance. A fixed reflecting prism beneath the disk directs the light from the disk along the optical axis when the zenith prism is at an angle of −90°.

Light from the zenith prism is focused onto the plane of an iris diaphragm by a lens. The iris aperture can be varied from 5 mm to 15 mm, allowing light from 3 solar diameters to 10°, respectively, to continue along the optical axis. The light then passes through two filterwheels, each with six 25.4 mm diameter holes spaced evenly about the wheel. The first filterwheel, designated FW#2, contains one open hole and five neutral density filters with nominal optical densities of 0.5, 1.0, 1.5, 2.0, and 2.5. These filters are used for O_3_, SO_2_, and NO_2_ measurements, while the open position is used for all other measurements.

The second filterwheel, designated FW#1, has two open holes for ultraviolet irradiance measurements, a ground-quartz diffuser for direct sun and quartz-halogen lamp measurements, a ground-quartz diffuser and neutral density filter combination for determinations of NO_2_, a film polarizer for zenith sky measurements, and a covered hole for dark signal tests. An 11.18 mm diameter fixed aperture after the filterwheels limits the field of view of the monochromator to *f*/6, and a lens after the aperture focuses the light onto the entrance slit of the monochromator.

The monochromator is a modified Ebert type with a 16 cm focal length and an aperture ratio of *f*/6. Light from the entrance slit passes through a lens, which corrects for the coma and astigmatic aberrations inherent in an Ebert monochromator, and is collimated by a spherical mirror onto the diffraction grating. After dispersion from the grating, a second reflection off the spherical mirror focuses the light onto the exit slit focal plane. The diffraction grating is holographic, has 1800 lines per millimeter on the Model MKII and 1200 lines per millimeter on the MKIV, and is coated with MgF_2_, as is the spherical mirror. The wavelength is adjusted by rotating the grating with a stepper motor which drives a micrometer acting on a lever arm. A 0.03 mm adjustment in the micrometer represents an approximately 0.1 nm wavelength change at the exit slit plane, while one motor step corresponds to approximately 0.006 nm.

The exit slit focal plane contains six slits, five for selecting the wavelengths for determining the amount of O_3_ and SO_2_: 306.3 nm, 310.1 nm, 313.5 nm, 316.8 nm, and 320.1 nm, and one for wavelength calibration. The same slits are used for determining NO_2_ at visible wavelengths: 431.4 nm, 437.3 nm, 442.8 nm, 448.1 nm, and 453.2 nm. A slotted cylindrical slitmask in front of the exit slit plane with eight positions serves as the wavelength selector. Six of the positions correspond to the six exit slits, one blocks the light for a dark signal measurement, and one exposes two slits so linearity of the detector can be determined. The nominal bandwidth, set by the exit slits, is 0.6 nm.

When measuring the amounts of O_3_ and SO_2_, the diffraction grating operates in the third order and is held fixed while the slitmask selects the wavelengths. For NO_2_, the grating operates in second order. Conversely, for calibration lamp and solar irradiance measurements, the angle of the diffraction grating is changed and the slitmask remains at a fixed position. For a MKII model, with the diffraction grating operating in third order and the first slit selected, the wavelength range for irradiance is 290 nm to 325 nm. The MKIV model extends the wavelength range by changing to second order and a different exit slit at 325 nm, making the operating range 286.5 nm to 363 nm.

Light from the exit slit passes through a quartz Fabry lens and a filter and is focused onto the cathode of a low-noise EMI 9789QA photomultiplier tube (PMT). The photon pulses from the PMT are amplified, discriminated, and divided by four before being transmitted to the counter. In the MKII model, the filter is NiSO_4_ sandwiched between two Schott UG-11 filters. The MKIV model has a third filterwheel, designated FW#3, instead of a single filter. One position contains only a UG-11 filter, another is blocked, the third a Schott BG-12 filter for NO_2_ measurements, and the fourth the NiSO_4_, UG-11 filter combination. This filter combination is used for measurements of O_3_, SO_2_, and irradiance at wavelengths shorter than or equal to 325 nm, while only the UG-11 filter is used at longer wavelengths. Thus, for wavelengths longer than 325 nm, the diffraction grating operates in second order rather than third order and a different exit slit and filter is used.

A separate computer is connected to the instrument by an RS-232C line and handles all data acquisition and control. Operation can be either semi-automatic, where the operator initiates a specific measurement, or fully-automatic, where the instrument follows a user-defined measurement schedule. An RCA COSMAC 18S601 microprocessor controls all internal low-level instrument functions. This includes positioning the diffraction grating and slitmask, turning calibration lamps on and off, and operating the stepping motors controlling the zenith prism, azimuth tracker, and filterwheels. The time and date are kept by a battery-protected internal board.

The data files are recorded on the computer. Each type of measurement has its own file structure; the structure for spectral scans of the irradiance from external sources, i.e., standard lamps or the sun, follows. The header contains the dead time of the photon counting system, the number of cycles over which counts were accumulated, the date and location, a voltage proportional to the temperature near the NiSO_4_ filter, and the dark count. When a standard lamp is scanned, the header also contains the lamp name. At each wavelength, the time, wavelength, micrometer step, and counts are recorded.

The count signal *S* is converted to observed photon rate *P*_0_ (photons per second) by the formula

$$P_{0}=\frac{4\left(S-D\right)}{0.2294\left(CY\right)},$$ (3.1)

where *D* is the dark count and *CY* is the number of cycles. The photons registered by the PMT are electronically divided by 4 prior to accumulation by the counter, and a single cycle is 0.2294 s in duration. Correcting for the dead time of the photon counting system yields the actual photon rate *P*_a_ (photons per second), given by

$$P_{0}=P_{a}\exp\left(-P_{a}\tau \right),$$ (3.2)

where *τ* is the dead time. This equation is solved iteratively for *P*_a_. The standard uncertainty in the photon rate is based on Poisson statistics of the number of photons detected, so that the standard uncertainty (one standard deviation estimate) in the total number of photons detected is given by

$$\sqrt{4\left(S-D\right).}$$ (3.3)

The relative standard uncertainty in *P*_a_ is then the reciprocal of the standard uncertainty given in Eq. (3.3).

The Brewer instrument was developed by Atmospheric Environment Service of Environment Canada and by the University of Toronto. It is manufactured and marketed by Sci-Tec Instruments, Inc., of Saskatoon, Saskatchewan, Canada. Of the more than 100 that have been manufactured, 77 have been incorporated in the World Ozone Network, and they are used at research facilities in more than 25 different countries. The AES operates a monitoring network of 10 sites in Canada, with a central calibration facility at the University of Toronto. The EPA monitoring network is administered by the University of Georgia, and currently has sites in Gaithersburg, Maryland; Boston, Massachusetts; Research Triangle Park, North Carolina; Atlanta, Georgia; and Bozeman, Montana. The instrument used at the Intercomparison was placed at the last site after the Intercomparison, and two more sites in this monitoring network are planned.

The wavelength of the monochromator in terms of micrometer steps was determined at the factory from the wavelengths of Hg emission lines. The wavelength registration of the monochromator is periodically checked and adjusted throughout a day by scanning the micrometer forward and backward about the 302.3 nm line from the Hg calibration lamp. The measured signal is compared with a reference signal to determine the step position which maximizes the correlation between the two signals. This is repeated, and adjustments are made in the step number, until the calculated step number falls within the acceptance limits.

The two networks use two different procedures for determining the spectral irradiance responsivity of their instrument from their spectral irradiance scale. The AES uses 1000 W, DXW-type quartz-halogen lamps operating in the horizontal position 40 cm above the diffuser. The lamp is housed in a custom enclosure with air drawn over the lamp, and baffling limits the light falling on the diffuser to the direct beam from the lamp. The current from a power supply is monitored through a calibrated shunt resistor by a voltmeter so that the operator can manually adjust the current as needed. The EPA uses the set of calibration lamps, housing, and power supply furnished by the manufacturer. These are 50 W quartz-halogen lamps mounted horizontally 5 cm above the diffuser in a housing and operated at a constant voltage of 12 V.

### 3.2 Biospherical Ultraviolet Spectroradiometer

The Biospherical SUV-100 B-007 Ultraviolet Spectroradiometer is a transportable version of spectroradiometers permanently installed at monitoring sites and performs spectral scans from 280 nm to 620 nm. It is housed in a weatherproof glass-fiber reinforced polyester resin molded enclosure. The enclosure is modular and can be separated into two sections for transport. The first section contains the foreoptics, monochromator, photomultiplier tube, and internal calibration sources. The second section contains the control electronics, main data acquisition system, auxiliary sensor system, and the Peltier cooler. Located up to 30 m away is the power supply for the calibration lamps and a computer for data acquisition and control.

**Fig. 3.2:**
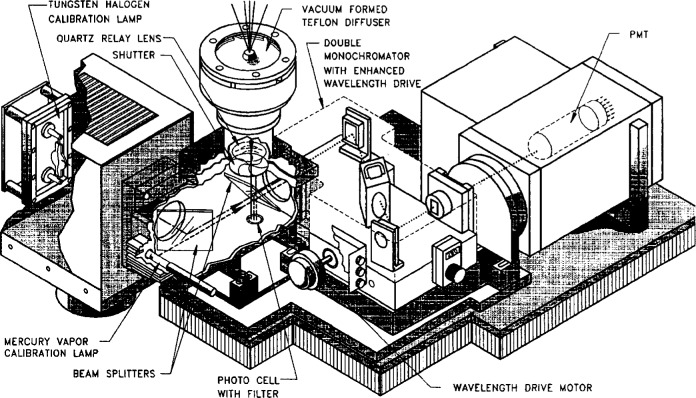
Cutaway diagram of the monochromator and collection optics of the Biospherical Instruments SUV-100 Ultraviolet Spectroradiometer.

A cutaway diagram of the monochromator and collection optics is shown in Fig. 3.2. The irradiance collector is a vacuum formed 2.16 cm diameter flat Teflon diffuser over quartz on the top of the instrument, and is heated by the system to minimize ice and snow buildup. Light from the diffuser passes through a quartz relay lens and a beamsplitter, where most of the light is directed into the monochromator, while some passes through to a stable photodetector filtered for response at UV-A wavelengths (315 nm to 400 nm). The beamsplitter also passes light from two calibration lamps, one a 45 W quartz-halogen lamp for spectral irradiance responsivity and the other a Hg emission lamp for wavelength. As an independent measure of UV-A irradiance, the photodetector enables system drifts to be detected and provides an indicator of changes in irradiance that may occur, for example, by cloud cover. The photodetector also monitors the output of the 45 W internal lamp.

The monochromator is an *f*/3.5, 0.1 m double-pass system with a 1200 lines per millimeter holographic grating blazed at 250 nm. The grating is driven by a stepping motor with a step size of 0.1 nm, and the bandwidth of the instrument is nominally 0.95 nm. The detector is a Hamamatsu R269 PMT with a quartz window, and the output signal is recorded in nanoamperes. The high voltage applied to the PMT is variable and is set for a specific spectral scan to obtain the maximum sensitivity. The PMT was selected for low noise at ambient temperatures and is mounted in a shielded housing and operated at 20 °C. The temperature of the monochromator is controlled to 32.5 °C and is typically stable to within 0.5 °C.

The data file from a spectral scan is in comma-separated format, with information about the conditions and operating parameters of the instrument in the header with nominal wavelength, current, and auxiliary channels following. There can be several items in each data file, each item corresponding to a set of fixed operating conditions, such as which lamps are on and the voltage of the PMT, and the parameters for the spectral scan.

A normal scan of solar irradiance consists of three items, each over a different wavelength range with different high voltages and wavelength intervals. Specifically, the first scan is over the range 280 nm to 315 nm with a 0.2 nm interval and the highest voltage, the next is over the range 280 nm to 380 nm with a 0.5 nm interval and a reduced voltage, and the last is over the range 280 nm to 620 nm with a 1.0 nm interval and the lowest voltage.

All nominal wavelengths are corrected before any further analysis of the data is performed. The internal Hg lamp is scanned several times each day, and offsets between the actual and measured wavelengths of the emission lines are calculated. A tangent method is used to calculate the wavelengths of the emission lines. Straight lines are fit to the seven points at wavelengths both longer and shorter than that of the peak signal. The intersection of these lines is the wavelength of the emission line. From the entire set of these scans during the observation period, i.e., during the Intercomparison, a piece-wise linear correction is developed for the nonlinearity in the wavelength transfer function of the monochromator. From each scan there is also an absolute offset applied to the nominal wavelength based upon the calculated wavelength of the 296.7 nm emission line of Hg. Thus, to correct the nominal wavelength from a scan of lamp or solar irradiance, the absolute offset is first applied, based upon the Hg scan done nearest in time to the scan, and then the non-linearity correction is applied. This results in a new data file with the corrected wavelength.

The spectral irradiance responsivity of the instrument is determined in a two-step process since the high voltage to the PMT is variable. The spectral irradiance scale is realized by 200 W, DXW-type quartz-halogen lamps mounted horizontally 50 cm above the diffuser. A lamp is located within a baffled enclosure that rests on top of the instrument, and a spectral scan is performed at a fixed high voltage. A spectral scan of the internal 45 W lamp is then performed at the same high voltage, and the ratio of the net signals obtained from the scans of the two lamps multiplied by the irradiance of the 200 W external lamp serves to calibrate the 45 W internal lamp. Spectral scans of this lamp at the voltages used for the solar scan items are performed several times a day and determine the spectral irradiance responsivity of the instrument.

The monitoring network established by the NSF and operated by Biospherical Instruments since 1988 has three sites in Antarctica, one in Ushuaia, Argentina, one in Barrow, Alaska, and one in San Diego, California. It is currently providing data to researchers studying the effects of ozone depletion on terrestrial and marine biological systems, in addition to being used to develop and verify models of atmospheric solar transmission and the impact of ozone depletion. Additional details on the data format, analysis techniques, and results from the monitoring activities can be found in Refs. [9, 10].

### 3.3 Smithsonian Ultraviolet Scanning Radiometer

**Fig. 3.3:**
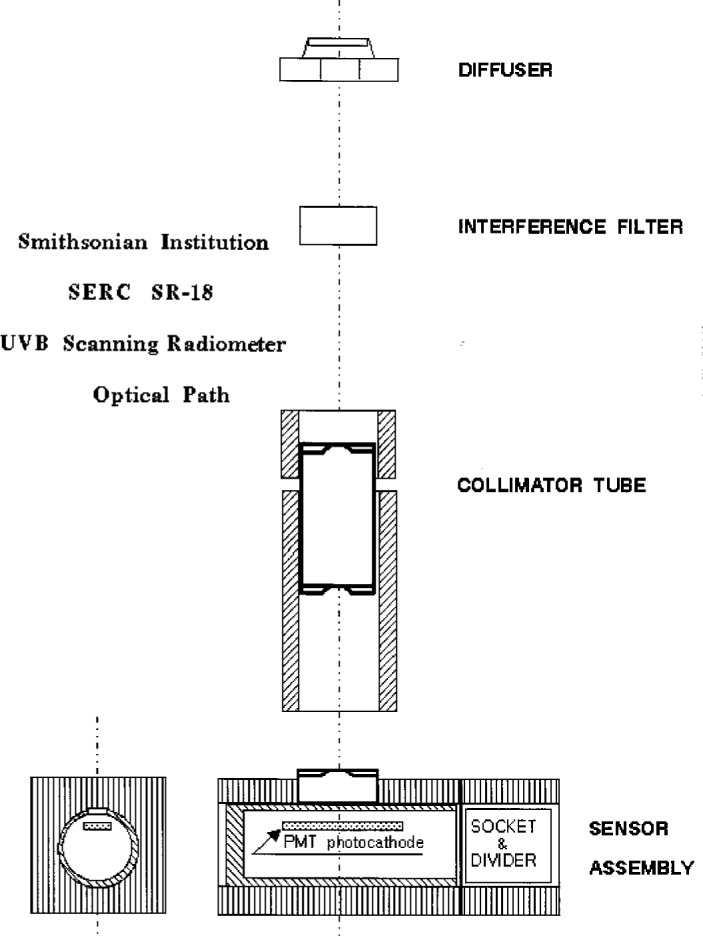
Schematic diagram of the optical components and path of a Smithsonian Ultraviolet Scanning Radiometer.

The SERC SR-18 Ultraviolet Scanning Radiometer measures irradiance at fixed wavelengths selected by 18 filters. The instrument consists of two units. The sensor head unit is a weather-proof housing with a 1.9 cm diameter Teflon cosine-corrected diffuser for light collection, a wheel with 18 interference filters with nominal 2 nm bandwidths mounted around the edge, a collimator, and a solar-blind Hamamatsu R-1657 PMT. A schematic diagram of the optical components and path is shown in Fig. 3.3. The PMT housing is temperature regulated at 20 °C with a thermostated thermoelectric system. The rotation rate of the filter wheel is 15 per minute. The filters have nominal center wavelengths from 290 nm to 324 nm at 2 nm intervals.

The sensor head is operated by an on-board microcontroller. The output current from the PMT is converted to a voltage by passing through a 10 MΩ resistor, filtered for electronic noise, and digitized by a precision monolithic 20-bit A/D converter. Voltages from all 18 filter wavelength channels, and two dark signal channels, are averaged for one minute, stored along with internal head temperature and control information, then transmitted over RS-422 twisted-pair wires to the data acquisition and control computer.

The sensor head unit is designed for field operation throughout the year. All components in the sensor head, except the PMT, are designed to operate without significant temperature effects over a temperature range of − 25 °C to 70 °C. A desiccant canister filled with indicating silica gel maintains low humidity within the sensor head, and the viewing port on this canister allows for pressure equilibration within the head.

The other radiometer unit is the external power supply for the sensor head. This power supply provides electrical power to both the head electronics and to the thermoelectric PMT temperature regulator. This unit is contained in a weatherproof housing.

For every minute that the radiometer is in standard operating mode, it transmits three lines of data to the data acquisition computer. The first line consists of the instrument identification and the current date and time. The time is that at the end of the minute which was just recorded. The third line consists of diagnostic information, including the temperature in the sensor head unit. The second line contains 20 channels of average voltages. Each channel includes an indicator character (A to T), a sign character (+ or −), and a seven digit decimal voltage value. The voltages from channels J and T are dark signals. The net signal for each channel is then the average of the dark signals subtracted from the voltage for that channel.

The present monitoring network of the SERC uses instruments similar to the ones described here, but with 8 filters having nominal 5 nm bandwidths at 5 nm intervals from 290 nm to 325 nm. The network has been operational since 1975 and had sites at the South Pole; Panama; Mauna Loa, Hawaii; Gainesville, Florida; Edgewater, Maryland; and Point Barrow, Alaska. Operational sites at present are the ones at Mauna Loa and Edgewater. The instruments described here will replace the current instruments as soon as they become operational.

The center wavelengths of the interference filters are nominally every 2 nm from 290 nm to 324 nm. To more accurately determine the actual center wavelengths, as well as additional information about the filters, SERC measures the transmittance of each filter every 0.5 nm with a Varian Cary 17D UV/VIS Spectrophotometer. The maximum transmittance *τ*_m_ of each filter is thus determined, along with the bandwidth Δ*λ* and actual center wavelength *λ*_0_. The bandwidth is the difference between the two wavelengths at which the filter transmittance is half its maximum value, and the center wavelength is the average of these two wavelengths. The actual center wavelengths, bandwidths, and maximum transmittances for all the filters of unit UC are given in Table 3.2. Additional details of the instrument, and results from the one located in Edgewater, are given in Ref. [11].

The spectral irradiance responsivity of a SERC instrument is determined by operating a calibrated 1000 W, FEL-type quartz-halogen lamp in the horizontal position centered 50 cm above the diffuser. The instrument is leveled and the lamp is positioned parallel to the diffuser. The line joining the centers of the diffuser and the lamp defines the optic axis. The net signal from each channel is divided by the irradiance of the lamp at the actual center wavelength to obtain the spectral irradiance responsivity for each channel. The angular response of the diffuser is determined with the same experimental setup. The instrument is rotated about an axis along the top and center of the diffuser so that the angle between the normal of the diffuser and the optic axis increases from 0° to 90°. Signals are recorded at evenly spaced angles. For unit UC, the angular response of the diffuser measured by SERC was within 3 % of that of the ideal cosine response over the entire range of angles.

## 4. Atmospheric Conditions

### 4.1 Meteorological

#### 4.1.1 Weather Summary

Weather conditions for the Intercomparison were ideal, with persistent clear skies throughout the period of the synchronized solar irradiance scans. Ironically, the Intercomparison was preceded by unseasonably cold and wet weather. As the outdoor instrument setup was underway on Sept. 21 (day 264), the first Arctic front of the season penetrated the central United States and brought the first snow to central Colorado. Fortunately, rapid storm development was followed by a persistent weather pattern that kept clear and dry conditions over Colorado for the duration of the outdoor measurements.

Conditions at the site of the Intercomparison are shown in Fig. 4.1, where the temperature, relative humidity, and atmospheric pressure are plotted as a function of time on each day of the synchronized solar scans. Unfortunately, some gaps occurred in the data. Days 266 and 267 had lower temperatures and higher humidity than days 269 and 270.

#### 4.1.2 Surface Conditions

On Sept. 20 (day 263), 2 days before the outdoor portion of the Intercomparison was to begin, an intense surface low over Saskatchewan brought the first major Arctic air outbreak of the season to the United States. By the afternoon of Sept. 21 (day 264) it brought cold, overcast conditions and the first snow of the season to Boulder. Frontal passage at the site of the Intercomparison occurred late in the morning of that day as some of the instruments were being set up. It was followed by about 12 h of driving wind, rain, and snow. Skies cleared by midnight under the influence of post-frontal high pressure. By the evening of Sept. 22 (day 265) the rapidly moving front was through south Texas. High pressure remained over Colorado and skies remained virtually cloud-free with low humidity for the remainder of the Intercomparison. Just as high pressure stagnated over the western United States, the surface low associated with this storm system also stalled to the east, keeping a large part of the United States in rain for much of the week.

Two weak surges of cold air entered the United States between Sept. 25 (day 268) and Sept. 28 (day 271), but both were halted in northeastern Wyoming by the high pressure and intense surface heating under the persistently clear skies to the south. Clouds associated with these fronts remained well north of Colorado. By Sept. 26 (day 269) the influence of high pressure (light winds and high stability) over several of the preceding days caused a buildup of particulates which created hazy conditions in the Boulder Valley. These conditions persisted on Sept. 26 to 28 (days 269 to 271). It was not until Sept. 29 (day 272) that the surface high pressure system in the west began to break down, initiating a return to more seasonal conditions. This unusual intransigence of the surface conditions was a direct consequence of an anomalous evolution of the upper air patterns, which is discussed in more detail in Appendix B.

### 4.2 Total Column Ozone

**Fig. 4.2:**
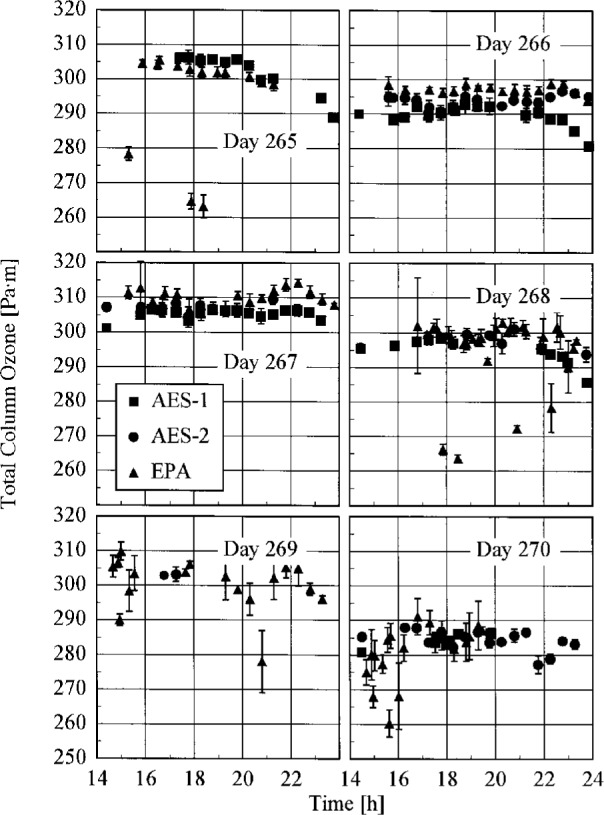
Total column ozone as a function of time on the days indicated in the panels as determined by the Brewer instruments indicated in the legend. The vertical bars are the standard uncertaintities of the values.

All three Brewer instruments measured total column ozone throughout the Intercomparison from measurements of the direct solar beam. The results are shown in Fig. 4.2, where the total column ozone is plotted as a function of time. The days are indicated in the panels, and the instruments are identified in the legend. The vertical bars are the standard deviation of each value. The total column ozone remained in the range 294 Pa·m to 314 Pa·m (290 matm·cm to 310 matm·cm) throughout most of the Intercomparison, and decreased to 284 Pa·m (280 matm·cm) on day 270. The total column ozone was particularly constant between instruments throughout days 266 and 270.

### 4.3 Broadband Measurements

A set of broadband radiometric instruments, listed in Table 1.1, were located on the test facility platform and made continuous measurements concurrently with the Intercomparison. Results from selected instruments are shown in Fig. 4.3, where the irradiance is plotted as a function of time for each instrument indicated in the panels. The solar pyranometer measured total horizontal irradiance in the band 280 nm to 3000 nm, while the ultraviolet radiometer measured the same quantity at ultraviolet wavelengths, from 280 nm to 320 nm. The normal-incidence pyrheliometer measured the irradiance from the solar beam in the band 280 nm to 3000 nm, while the infrared pyrgeometer measured the total horizontal irradiance at infrared wavelengths, from 3 μm to 50 μm.

The clear, cloudless skies on days 266 to 268 are evident in Figs. 4.3(a), 4.3(b), and 4.3(c). The irradiances measured by the pyranometers and pyrheliometer are smooth throughout the days, with peaks at solar noon, approximately 19:00 h. The irradiance measured by the pyrgeometer also increased smoothly during the daylight hours. Cloudiness on day 269, especially around solar noon, is indicated in Fig. 4.3(d) by the sharp decreases in irradiance measured by the pyranometers and pyrheliometer, and by the increases in irradiance measured by the pyrgeometer, which senses the warmer clouds. This cloudiness continued on day 270, as shown in Fig. 4.3(e), especially in the late afternoon, and in addition the atmospheric turbidity caused a reduction in the maximum irradiances compared to those on the clear days.

**Fig. 4.4:**
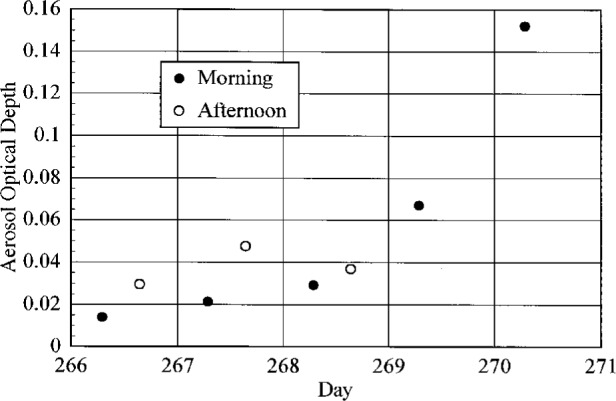
Aerosol optical depth at 500 nm in both the morning and afternoon as a function of day during the Intercomparison.

The aerosol optical depth was measured at seven wavelengths in the visible and near-infrared regions of the spectrum using a multi-filter rotating shadowband radiometer. The aerosol optical depth at 500 nm is shown in Fig. 4.4 as a function of day over the duration of the synchronized solar scans. The values obtained from both morning and afternoon Langley regressions are shown. From Fig. 4.4, it is apparent that the turbidity of the atmosphere increased nearly monotonically during the time interval.

## 5. Instrument Characterizations

The spectroradiometers were characterized for the parameters which most affect their ability to accurately measure solar ultraviolet irradiance, and which did not require elaborate experimental equipment or techniques. This latter requirement was particularly important because of the time and facility limitations imposed by working at a remote location. The instrument characterization experiments were limited to ones that used a minimal amount of equipment that was easy to transport and set up, and used existing computer programs for operating the instruments. Therefore, the slit-scattering function, stray-light rejection, wavelength accuracy, and spectral irradiance responsivity were determined, while other possible experiments such as linearity and cosine response were not. All of the characterizations were performed indoors in the concrete building prior to deploying the instruments on the pads, and in addition the spectral irradiance responsivity of each instrument was determined twice outdoors on the pads.

The basis for the characterizations is the simplified measurement equation, given by

$$S\left(\lambda_{0}\right)=\int E\left(\lambda \right)\;R\left(\lambda_{0},\;\lambda \right)d\lambda ,$$ (5.1)

where *λ* is the wavelength, *λ*_0_ is the wavelength setting of the monochromator, *S*(*λ*_0_) is the output signal, *E*(*λ*) is the spectral irradiance of the source, and *R*(*λ*_0_, *λ*) is the spectral irradiance responsivity function of the instrument. Further, the spectral irradiance responsivity function is given by

$$R\left(\lambda_{0},\lambda \right)=R\left(\lambda \right)z\left(\lambda -\lambda_{0}\right),$$ (5.2)

where *R*(*λ*) is the irradiance response function and *z* (*λ* − *λ*_0_) is the dimensionless slit-scattering function. Combining Eqs. (5.1) and (5.2) yields

$$S\left(\lambda_{0}\right)=\int E\left(\lambda \right)\;R\left(\lambda \right)z\left(\lambda -\lambda_{0}\right)\;d\lambda .$$ (5.3)

The spectral irradiance *E* (*λ*) can either be known, i.e., a standard lamp, or the desired result, i.e., measurements in the ultraviolet region of the solar spectrum. The response function varies slowly with wavelength, and indicates the sensitivity of the instrument to irradiance at a given wavelength. Conversely, the slit-scattering function is a rapidly varying function of wavelength, is ideally independent of *λ*_0_ and triangular in shape, and determines the full-width-at-half-maximum (FWHM) bandwidth Δ*λ* of the instrument.

### 5.1 Slit-Scattering Function and Stray-Light Rejection

#### 5.1.1 Introduction

The discussion of the characterization of the instruments begins in terms of their response to light at 325.029 nm from a HeCd laser because it is conceptually the simplest case and yet yields much valuable information. The measurement equation given by Eq. (5.3) becomes considerably simplified when the source is monochromatic, such as a laser, at wavelength *λ*′. Then, *E* (*λ*) = *E*_0_δ(*λ* − *λ*′), and

$$S\left(\lambda_{0}\right)=E_{0}\;R\left(\lambda^{\prime }\right)\;z\left(\lambda^{\prime }-\lambda_{0}\right).$$ (5.4)

Thus, the wavelength dependence of the irradiance response function does not enter into the analysis since the light is monochromatic, and normalizing the signals recorded from the spectral scan to the maximum signal gives the slit-scattering function with a peak value of unity. When the slit-scattering function is determined in this manner, *λ*_0_ is the independent variable while *λ*′ remains constant. Performing a change of variables so that *λ* is the independent variable and *λ*_0_ is constant yields *z* (*λ*_0_ − *λ*). Therefore, the stray-light rejection at wavelengths shorter than that of the peak signal in a plot of peak-normalized signal as a function of monochromator wavelength is actually the stray-light rejection of the instrument at longer wavelengths.

#### 5.1.2 Experimental Procedure

An Omnichrome Model 3056 HeCd laser with a single line at 325.029 nm and a nominal power of 5 mW was placed on a table in a darkened room which was almost completely isolated from ambient light. The output of the laser was first directed through a prism (to remove amplified spontaneous emission, or bore glow), then through a beamsplitter (to monitor the output of the laser), and finally across the room and reflected off a mirror onto the diffuser of the instrument. A Si photodiode monitored the beam and its output was amplified, converted to voltage, and recorded by a digital voltmeter. The variation in the laser output over the course of a spectral scan was always less than 1 %. The beam diameter was approximately the same diameter as the diffusers.

High-resolution spectral scans were performed near 325 nm to obtain the bandwidth of the instrument, centroid of the line, and shape of the slit-scattering function near its peak. These scans covered the wave-length range 320 nm to 327 nm for the AES instruments, and up to 330 nm for the EPA instrument, at 0.1 nm increments. One neutral-density filter on FW#2 was in the internal optical path for the AES-1 instrument, while two scans were made with two different filters on FW#2 for the AES-2 and EPA instruments. The wavelength range for the NSF instrument was 320 nm to 330 nm at 0.1 nm increments. Lower-resolution spectral scans were performed across wider wavelength ranges of the instruments to obtain the full slit-scattering function. The wavelength ranges were 290 nm to 327 nm for the AES instruments, the first at 0.25 nm increments and the second at 0.2 nm increments, 286.6 nm to 363 nm at 0.2 nm increments for the EPA instrument, and 270 nm to 400 nm at 1.0 nm increments for the NSF instrument. During these scans, there were no neutral-density filters in the internal optical paths of the Brewer instruments. For the SERC instrument, the signals were measured for 3 min. Finally, a lower-resolution scan was performed with the laser beam blocked to check for stray light from sources other than the laser. There were no signals greater than the dark signal in any of the instruments.

#### 5.1.3 Data Analysis

While not important for spectral scans of laser lines, because the light is monochromatic, background subtraction is important for spectral scans of lamp emission lines because of the underlying continuous emission from these lamps. To maintain consistency, background subtraction was also performed on spectral scans of laser light. The background signal is described by a linear fit of the signals at wavelengths that differ by 1.5 bandwidths from the wavelength of the peak signal. For unresolved multiple lines in emission lamps, the factor is increased from 1.5 to 2.0. The signals and wavelengths for the first five consecutive data points that lie outside this range are averaged and fit with a straight line to yield background signal as a function of wavelength. This fit is subtracted from the signals within the range. There is obviously an interplay between the background subtraction and the bandwidth, but a consistent bandwidth can be obtained after only one or, at most, two iterations between background subtraction and the bandwidth calculation.

The bandwidth of the instrument is defined here as the FWHM from a high-resolution spectral scan of a laser line or a singlet lamp emission line. For a lamp emission line, the signal is converted to irradiance, as detailed below. Linear interpolation is used to find the wavelengths at which the signal is one-half that of the peak. The bandwidth is then the difference between these two wavelengths.

The centroid method is used as the best estimate of the wavelengths of laser lines and lamp emission lines since the instruments perform spectral scans at discrete wavelengths. The centroid *C* from a high-resolution scan is given by

$$C=\sum_{i}S_{i}\lambda_{i}/\sum_{i}S_{i},$$ (5.5)

where *i* indexes the signals *S_i_* and wavelengths *λ_i_*, respectively, within the specified range, either 1.5 or 2.0 bandwidths on either side of the wavelength of the peak signal. The irradiances *E_i_* can be substituted for the signals *S_i_* in Eq. (5.5).

The presence of different filters in the optical path of the Brewer instruments complicates the analysis of their spectral scans. The neutral density filters on FW#2 are useful for preventing saturation of the PMT, while the switch of filters on FW#3 between the NiSO_4_, UG-11 combination and only UG-11 at 325 nm for the EPA instrument causes a discontinuity in the signal at that wavelength.

The optical densities of the neutral-density filters at 325 nm were determined by finding the common wavelengths at which signals were measured for scans both with and without filters. The relation between the signal with a filter, *S*_f_, and the signal at the same wavelength without a filter, *S*_0_, is given by

$$S_{f}=S_{0}\times 10^{-D},$$ (5.6)

where *D* is the optical density. Therefore, the optical density is given by

$$D=\log_{10}\left(S_{0}/S_{f}\right).$$ (5.7)

Performing this calculation for the non-saturated signals at each wavelength and averaging the values yields the optical density of the filter.

For the high-resolution scans, normalization of the signals by the peak signal was straight-forward since there was no saturation of the signal. For the low-resolution scans, however, the normalization is more complicated since the signals from the Brewer instruments saturated near 325 nm. Therefore, the peak signal from the high-resolution scan and the optical density of the filter were used in Eq. (5.6) to calculate the peak signal for the scan without the neutral-density filter. For the AES-1 instrument, only one peak signal was calculated since only one neutral-density filter had been used, while for the AES-2 instrument the peak signal was the average of the values calculated with the two neutral-density filters. This second procedure was also used with the EPA instrument, with the added feature that peak signals were calculated for wavelength regions both greater than and less than 325 nm and then applied to the signals in those regions.

The peak signal obtained in the high-resolution scan was used to normalize the signals from the low-resolution scan for the NSF instrument since there was no saturation. The peak signal for the SERC instrument was not as readily known since there is no filter centered at 325 nm. Therefore, the peak signal for each filter was obtained from the measured signal of the filter centered at the longest wavelength that did not saturate. These peak signals were calculated by dividing the measured signal from the filter centered at 320.23 nm by the transmittance of that filter at 325 nm and multiplying by the peak transmittance of each filter.

#### 5.1.4 Results and Discussion

The bandwidths of the instruments and the centroids of the laser line are most useful when compared to those values obtained from the scans of a Hg lamp. Therefore, the results from these determinations are shown in Figs. 5.3 and 5.4 in the next section. The bandwidths at 325 nm are close to the nominal values, 0.6 nm for the Brewer instruments and 0.95 nm for the NSF instrument.

**Fig. 5.1:**
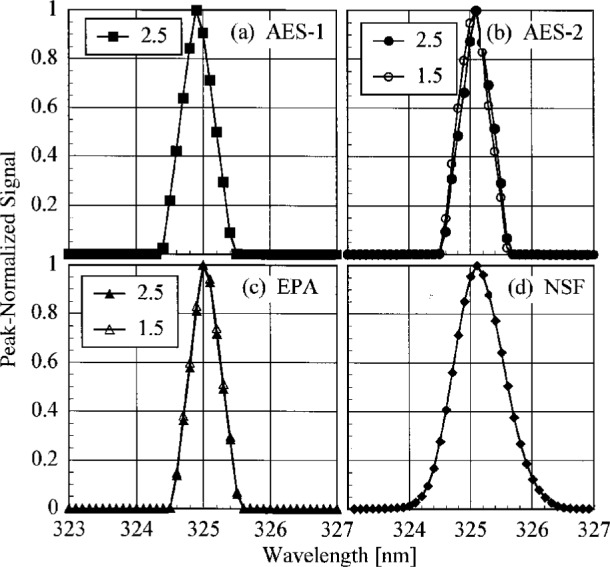
Peak-normalized signal as a function of wavelength from high-resolution spectral scans of the 325.029 nm line from a HeCd laser for the instruments indicated in each panel, demonstrating the slit-scattering function for each. The nominal optical density of the neutral density filter used with the first three instruments is indicated in the legends.

**Fig. 5.2:**
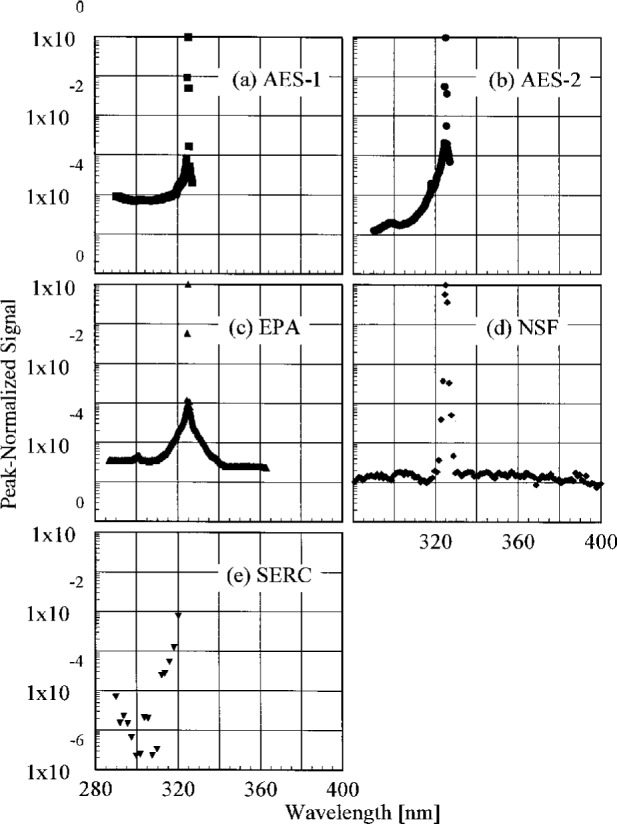
Peak-normalized signal as a function of wavelength from low-resolution spectral scans of the 325.029 nm line from a HeCd laser for the instruments indicated in each panel, demonstrating the slit-scattering function and stray-light rejection for each.

The slit-scattering functions are shown in Figs. 5.1 and 5.2, from high-and low-resolution scans, respectively, where the peak-normalized signal is plotted as a function of wavelength. From Fig. 5.1, the slit-scattering functions of the Brewer instruments are nearly triangular and symmetric about the peak wavelength, while that for the NSF instrument is more Gaussian. The stray-light rejection of each instrument, from Fig. 5.2, is the peak-normalized signal at the shortest wavelengths. The stray-light rejection of approximately 10^−4^ is reasonable for the Brewers since they are single-grating instruments. For the NSF instrument, however, the magnitude of the stray-light rejection, 10^−5^, is greater than expected for a double monochromator, probably from the limited dynamic range possible with the PMT operating in current mode. As will be discussed below, the actual stray-light rejection of this instrument is better than what is shown in Fig. 5.2, which demonstrates that an accurate determination of the stray-light rejection requires both a source with sufficient power and a detector with sufficient dynamic range. The stray-light rejection of the SERC instrument, approximately 10^−5^, is also reasonable, although there is evidence for a light leak at the shortest wavelengths.

### 5.2 Wavelength Accuracy

#### 5.2.1 Introduction

Characterizing the instruments in terms of their response to light from a Hg emission line lamp is somewhat more complex than was the case for a HeCd laser both because there is a continuum in addition to the lines and because there can be unresolved multiple lines. However, it is useful because it yields information about the wavelength repeatability and accuracy of the instruments and about the bandwidth at several wavelengths. The wavelength accuracy is especially important in the UV-B region of the solar spectrum (280 nm to 315 nm) because the irradiance at the Earth’s surface changes rapidly with wavelength, so a small uncertainty in wavelength translates into a large uncertainty in irradiance.

A distinction needs to be made between wavelength calibration and wavelength registration, both of which affect the wavelength accuracy. The wavelength calibration is the relation between the motor steps that determine the grating angle and the monochromator wavelength, and is determined from the emission lines of a Hg lamp. The wavelength calibration is in general a non-linear function of motor steps. The wavelength registration is a fixed offset of motor steps from a known position, which is provided by the 302.3 nm line of Hg for the Brewer instruments and by the 296.7 nm line for the NSF instrument.

The wavelengths of emission lines from gas lamps are known to high accuracy. However, the relative intensities of these lines change with lamp and operating condition. Therefore, an Oriel Model 6035 Hg emission lamp was used because of recent measurements of the relative intensities of the lines from this particular model of lamp [12, 13].

#### 5.2.2 Experimental Procedure

In the laboratory, the Hg emission lamp was placed horizontally and as close as practical over the diffuser of the instrument. The lamp was warmed up for 10 min, the current was set at 10.0 mA, and a spectral scan was performed by the instrument. A black cloth was placed over, but not on, the lamp both to reduce background light and for safety considerations.

All the Brewer instruments performed spectral scans over their entire operating ranges at 0.05 nm increments. The NSF instrument performed spectral scans of each emission line at 0.05 nm increments. A second scan was performed outdoors since there had been a problem with the first indoor scan of the line at 435.833 nm.

#### 5.2.3 Data Analysis

Because of the background emission continuum, the signals from scans were converted to irradiance using a simplified version of Eq. (5.3), namely

$$S\left(\lambda_{0}\right)=E\left(\lambda_{0}\right)R\left(\lambda_{0}\right).$$ (5.8)

The spectral irradiance responsivity *R*(*λ*_0_), determined from other measurements, was fit with a natural cubic spline to the wavelengths of the scan, and then the signal was divided by this responsivity to obtain irradiance. Background subtraction and calculation of the centroid and bandwidth for each line were performed as detailed in the preceding section. Only the bandwidths for single lines were taken to be indicative of the bandwidth of the instrument at that wavelength. The actual centroids of the lines were calculated from the wavelengths and relative intensities of the lines for that particular model of Hg lamp.

Unfortunately, the 0.05 nm increment for the NSF instrument proved to be too small, as the data points were “paired” in wavelength due to the step resolution of the diffraction grating motor. Therefore, the data at every other wavelength in the scans was used for the calculations. The results depended somewhat upon the starting wavelength, so that was chosen based upon the one that yielded line centroids closest to the actual ones.

#### 5.2.4 Results and Discussion

**Fig. 5.3:**
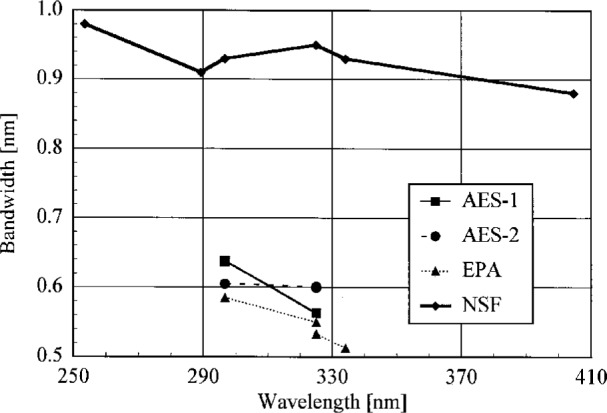
Bandwidth as a function of wavelength for the instruments indicated in the legend from high-resolution spectral scans of singlet Hg emission lines and the 325.029 nm line from a HeCd laser.

**Fig. 5.4:**
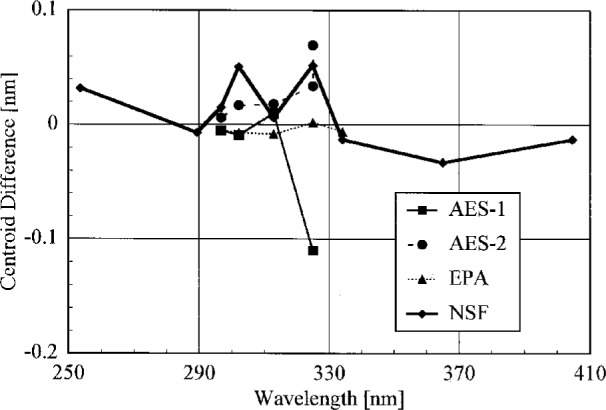
Centroid difference between the calculated and actual values for the instruments indicated in the legend from high-resolution spectral scans of Hg emission lines and the 325.029 line from a HeCd laser demonstrating the wavelength accuracy of each instrument.

The bandwidths calculated from the singlet Hg lines and the HeCd line are plotted in Fig. 5.3 as a function of wavelength. Likewise, the differences between the calculated and actual centroids of the Hg and HeCd lines are plotted in Fig. 5.4 as a function of wavelength. The instruments are indicated in the legends.

To obtain centroids of multiple lines that were consistent with those determined for single lines, it was very advantageous to use a Hg lamp for which the relative intensities of the lines were accurately known. The centroids of the Hg emission lines properly give the wavelength repeatability of the instrument since all of the instruments use these lines in their wavelength calibrations. Therefore, from Fig. 5.4, the wavelength repeatability of the Brewers was better than 0.02 nm, while for the NSF instrument it was slightly less than 0.06 nm. An assessment of the wavelength accuracy is best done with the line from the HeCd laser since this wavelength is not used in the wavelength calibration. The measured centroid of the HeCd line differs from its actual value by 0.11 nm, 0.07 nm, 0.002 nm, and 0.05 nm for the AES-1, AES-2, EPA, and NSF instruments, respectively. The first two values are significantly greater than those obtained from the Hg emission lines, while the third is less and the fourth is comparable. Therefore, considering both the Hg and HeCd centroids, the wavelength standard uncertainty for all the instruments is taken to be at most 0.1 nm. These values will be used below in the uncertainty analysis of the instrument spectral irradiance responsivity.

The bandwidths of all the instruments show a general decrease with increasing wavelength. This is in contrast to the increase expected from the simple grating equation. Therefore, additional elements along the optical paths of the instruments, such as lenses, are causing the observed dependence of bandwidth on wavelength.

### 5.3 Spectral Irradiance Responsivity

#### 5.3.1 Introduction

Determining the spectral irradiance responsivity (hereafter termed simply the responsivity) of the instruments, both with a NIST working standard lamp and with the standard lamps of the participants, was for several reasons the most important aspect of the Intercomparison. Comparing the responsivities obtained with the NIST standard lamp with those from the participants’ lamps showed the agreement between the spectral irradiance scales. Comparing responsivities obtained in the laboratory and outside demonstrated the translational and temporal stability of the instruments. Finally, using the responsivity of each instrument determined by the NIST standard lamp to calculate the solar irradiance removes discrepancies among the participants’ spectral irradiance scales from field measurement intercomparisons between instruments.

All but one of the participants brought spectral irradiance standard lamps to determine the responsivity of their instrument. The NIST working standard lamp for spectral irradiance was a 1000 W, FEL-type quartz-halogen lamp calibrated in the horizontal position, i.e., the long axis of the lamp is horizontal [14]. Both the responsivity of an instrument and the solar irradiance is determined from the simplified measurement equation

$$S\left(\lambda_{0}\right)=E\left(\lambda_{0}\right)\;R\left(\lambda_{0}\right).$$ (5.9)

A standard lamp has a known spectral irradiance *E*(*λ*_0_), so dividing the signals *S*(*λ*_0_) from a spectral scan by the irradiance yields the responsivity *R*(*λ*_0_). Given this responsivity for the instrument, the solar irradiance *E*(*λ*_0_) is given by dividing the signals *S*(*λ*_0_) from a spectral scan of this irradiance by the responsivity *R*(*λ*_0_).

#### 5.3.2 Experimental Procedure

The responsivity was determined in the concrete building for every instrument except that from SERC. An area in the building was surrounded by black cloth, including the ceiling, and the outside windows were also covered with black cloth. The room lights were turned off during the measurements.

There were two independent sets of spectral irradiance standard lamps for the AES instruments: 50 W quartz-halogen lamps supplied by Sci-Tec, Inc. housed in an enclosure and 5 cm above the diffuser, designated 321 and 322; and 1000 W, DXW-type quartz-halogen lamps in a custom enclosure 40 cm above the diffuser, designated S-734, S-789, and U-4. The EPA also had a set of 50 W quartz-halogen lamps supplied by Sci-Tec, designated 296 and 297. The NSF instrument used a 200 W, DXW-type quartz-halogen lamp, supplied and calibrated by Optronics Laboratories, in a custom enclosure 50 cm from the diffuser, designated M-761. All of the enclosures allowed the participants to determine the responsivity in the normal orientation of the instrument both indoors and outdoors. The responsivity of the SERC instrument had been determined at the home laboratory with a 1000 W FEL-type quartz-halogen lamp.

The spectral irradiance of the 1000 W, FEL-type NIST standard lamp, designated OS-27, had been determined in the horizontal position as described in Ref. [14]. The tripod system used in this determination was also used at the Intercomparison. The technique is briefly described here, while more details about the experimental procedure and uncertainty analysis are given in Ref. [14]. A kinematic lamp mount, with tilt and translation stages, was attached to a tripod, and a HeNe laser was attached to the tripod 60 cm above the lamp mount with a tilt stage for alignment with the instrument diffuser. The optical axis was iteratively set by centering the laser beam on the diffuser and by retroreflecting the beam from a glass slide placed over the diffuser. After this procedure, the laser was not adjusted further. The lamp jig was adjusted with the stages to center the jig perpendicular to the optical axis, as defined by the laser beam, and 50.0 cm from the diffuser. A constant current of 7.9 A was passed through the lamp by a power supply system described in more detail in Ref. [15]. For all determinations of instrument responsivity, the first spectral scan was performed with a 3.5 cm wide shutter halfway between the lamp and the diffuser to block the direct beam from the lamp and thereby measure the diffuse signal. The succeeding spectral scan(s) did not use the shutter and therefore measured the total signal.

The AES instruments presented a challenge for alignment because the quartz dome prevented direct access to the diffuser. A 40 cm long cylinder from the participant’s lamp enclosure that fit around the diffuser assembly supported the glass slide used to retroreflect the laser beam. The laser beam was centered on the diffuser by adjusting the position of the laser so that all the beam paths through the dome converged on the same position on the diffuser. The 50.0 cm distance between diffuser and lamp jig was referenced from the ring around the dome. While all of these techniques allowed the lamp to be aligned, they also increased the uncertainties associated with the lamp alignment. Prior to the spectral scans of standard lamps, the wavelength of the monochromator was set using the usual routine from a scan of the internal Hg lamp. Spectral scans of standard lamps were performed from 290 nm to 325 nm at 3.5 nm increments with both increasing and decreasing wavelength. One such scan was generally done with the shutter in place, and two or three more without the shutter.

The quartz dome was removed during lamp alignments for the EPA instrument. A glass slide was placed on the diffuser for the laser beam retroreflection, while the laser beam was centered on the diffuser with the help of a ruler placed on the diffuser. The distance from the lamp to the center of the diffuser was set at 50.0 cm, and the dome was replaced after the alignment was completed. As with the AES instruments, the wavelength of the monochromator was set using the internal Hg lamp. Spectral scans of standard lamps were performed from 286.5 nm to 363 nm at 0.5 nm increments with increasing wavelength, one with the shutter and one without.

Aligning the lamp with the NSF instrument followed the same procedure described above for the EPA instrument. Spectral scans of standard lamps were performed from 280 nm to 400 nm at both 5.0 nm and 1.0 nm increments with increasing wavelength, one with the shutter and one without.

A glass slide for retroreflection was placed on the ring surrounding the diffuser of the SERC instrument, while a ruler was used to help center the laser beam on the diffuser. For the first determination of responsivity outdoors, the 50.0 cm distance between the lamp and diffuser was mistakenly measured from the top of the ring, while it was correctly measured from the diffuser for the second determination. The top of the ring was 1.2 mm above the diffuser, and the irradiance of the lamp was corrected for the distance. Signals from the instrument were collected for 10 minutes both with and without the shutter in place.

The responsivities of the AES, EPA, and NSF instruments were determined once indoors, while the responsivities of all the instruments were determined twice outdoors. The same NIST standard lamp, power supply, tripod support, and alignment procedure were used in all the determinations of responsivity. The outdoor measurements were performed at night so there was no solar contribution to the signal. Fortunately, there was no problem with insects being attracted to the light of the lamp since the nights were cool.

A schedule of the spectral scans of standard lamps is given in Table 5.1, along with the corresponding instrument temperatures. Lamps OS-27 and F-45 were standard lamps from NIST, the latter having a frosted envelope. This lamp performed poorly during the indoor spectral scans and so was not used outdoors. However, spectral scans taken with this lamp and the EPA instrument both with and without the quartz dome over the diffuser showed that the dome decreased the responsivity of the instrument by approximately 6 %, independent of wavelength. The spectral scans on days 262 to 264 were performed indoors, while the remainder were outdoors. Most of the lamps were described previously, except for GS-918, which was a 1000 W, FEL-type used by the EPA network, and the other lamps designated only with numbers, which were 50 W lamps supplied by Sci-Tec.

#### 5.3.3 Data Analysis

In most cases, the signal used to determine the responsivity of the instrument from the NIST standard lamp was the direct signal, given by the difference between the total signal (measured without the shutter) and the diffuse signal (measured with the shutter). For those instruments that had multiple readings at each wavelength of the scan (AES-1 and AES-2), the standard uncertainty in the signal was the standard deviation of the mean of the signals, while for those that had only one reading and counted photons (EPA), Poisson statistics were used to determine the standard uncertainty given by the square root of the number of photons. For spectral scans of participants’ lamps, the total signal was used to determine the responsivity since a shutter was not used to measure the diffuse signal.

For the AES-1 instrument, the responsivity using the NIST standard lamp was always calculated from the total signal since the diffuse signal was the same as the dark signal, indicating that there was no measurable diffuse signal. The standard uncertainty in the total signal was the standard deviation of the mean of the measured signals at each wavelength since several scans were performed. However, for the AES-2 instrument the responsivity using the NIST standard lamp was always calculated from the direct signal since there was a measurable diffuse signal, probably from reflections from the newer paint on the instrument. The standard uncertainties of the total and diffuse signals, from the standard deviations of the mean, were propagated through to give the standard uncertainties of the direct signals. The responsivity of the EPA instrument was calculated from the direct signal for the scan indoors, and from the total signal for the two outdoor scans because the diffuse signals were the same as the dark signal. The standard uncertainties were determined from Poisson statistics since only one measurement was taken at each wavelength, and the uncertainties were propagated through for the direct signals.

Spectral scans of external lamps by the NSF instrument consisted of a scan with the internal shutter closed followed by a scan with the shutter open. The dark signal is the average of the signals with this shutter closed, and the net signal for a given spectral scan is the difference between the signal measured with the shutter open and the dark signal. The standard deviation of the dark signal, multiplied by $\sqrt{2}$, is taken to be the standard uncertainty of the net signal. The responsivity using the NIST standard lamp was always calculated from the direct signal. For the SERC instrument, the responsivity using the NIST standard lamp was always calculated from the direct signal. The standard uncertainty for each average signal was the standard deviation of the mean of the measured signals, and was propagated through to the direct signal.

The uncertainty analysis for the responsivities follows the approach given in Ref. [14], so the details will not be repeated here. Components of uncertainty arise from the calibrated spectral irradiance of the standard lamp, the goniometric distribution of irradiance, the current through the lamp, the wavelength of the instrument, the alignment of the lamp, and the measured signal. All of these components are included in the standard uncertainty of responsivity determined with the NIST standard lamp, while only the standard uncertainty of the signal is included for responsivities determined with the participants’ lamps since the other components were not determined for these lamps.

The standard uncertainties from these components are listed in Table 5.2, while Table 5.3 lists the corresponding relative standard uncertainties, at selected wavelengths. The relative standard uncertainties in the spectral irradiance and the goniometric distribution of the lamp were determined when it was calibrated at NIST. The standard uncertainty in the current is the root-sum-square of uncertainties from the resistance of the shunt and the voltage measured by the voltmeter. The standard uncertainty in the wavelength of each instrument was determined in the previous section. The standard uncertainties in the alignment of the lamp are those arising from centering the diffuser and lamp jig on the optical axis, aligning the diffuser and lamp jig perpendicular to the optical axis, and setting the distance from the diffuser to the lamp jig. The standard uncertainty in the signals was calculated for each instrument as described previously.

The relative standard uncertainties are conveniently grouped according to their origin. Those arising from random effects are the signals, current through the lamp, wavelength of the instrument, and lamp alignment. The latter three are combined together and designated the Lamp (*R*) relative standard uncertainty, while the first is simply the Signal (*R*) relative standard uncertainty. Those arising from systematic effects are the spectral irradiance and goniometric distribution for the lamp. These are combined and designated the Lamp (*S*) relative standard uncertainty. The Lamp relative standard uncertainties are listed in Table 5.3. These designations are important when comparing responsivities. For example, the relative standard uncertainty in the ratio of the responsivities determined by the NIST standard lamp and by a participant’s lamp include components of uncertainty arising from both random and systematic effects. However, the relative standard uncertainty in the ratio of two responsivities determined by the NIST standard lamp includes only components of uncertainty arising from random effects.

#### 5.3.4 Results and Discussion

The spectral irradiance of each participant’s lamp is based upon the spectral irradiance scale used by that participant’s monitoring network. These scales are based upon calibrated lamps supplied by different manufacturers. A comparison between these scales and the NIST spectral irradiance scale is very important to assess the accuracy of the participants’ scales. This comparison is obtained by the ratio of the responsivity using the NIST standard lamp to the responsivity using the participant’s standard lamp(s). The simplified measurement equation for the signal using the NIST standard lamp is

$$S_{N}\left(\lambda_{0}\right)=E_{N}\left(\lambda_{0}\right)\;R_{N}\left(\lambda_{0}\right),$$ (5.10)

where the subscript N indicates the NIST standard lamp. Likewise, for a participant’s standard lamp, indicated by the subscript P,

$$S_{P}\left(\lambda_{0}\right)=E_{P}^{\prime }\left(\lambda_{0}\right)\;R_{P}\left(\lambda_{0}\right).$$ (5.11)

In Eq. (5.11), *E*′_P_(*λ*_0_) is the spectral irradiance assigned to the standard lamp by the participant. Taking the actual responsivity to be *R*_N_(*λ*_0_), Eq. (5.11) can be rewritten as

$$S_{P}\left(\lambda_{0}\right)=E_{P}\left(\lambda_{0}\right)\;R_{N}\left(\lambda_{0}\right),$$ (5.12)

where *E*_P_(*λ*_0_) is the actual irradiance of the participant’s standard lamp based upon the NIST scale. Equating Eqs. (5.11) and (5.12) and rearranging yields

$$\frac{R_{N}\left(\lambda_{0}\right)}{R_{P}\left(\lambda_{0}\right)}=\frac{E_{P}^{\prime }\left(\lambda_{0}\right)}{E_{P}\left(\lambda_{0}\right)}.$$ (5.13)

Therefore, the ratio of the spectral irradiance assigned by the participant to the actual spectral irradiance based upon the NIST scale is the ratio of the responsivity determined with the NIST standard lamp to that determined with the participant’s standard lamp.

**Fig. 5.5:**
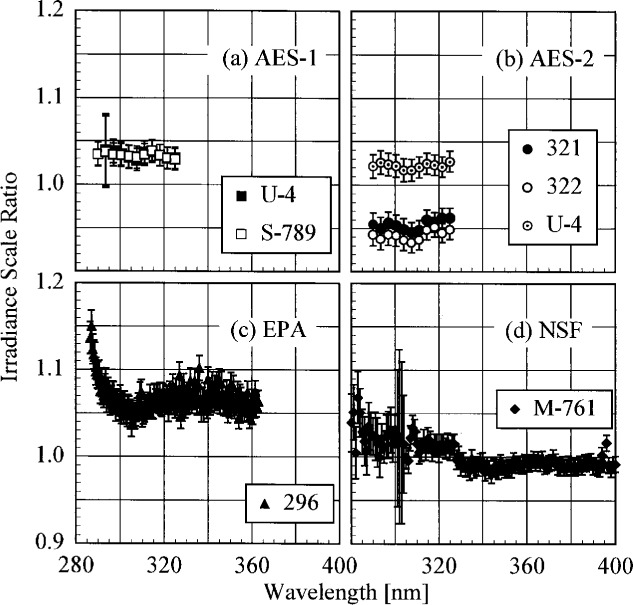
Ratio of the participants’ spectral irradiance scales to the NIST spectral irradiance scale as a function of wavelength from spectral scans performed indoors. The instruments are indicated in each panel, the participants’ lamps are indicated in the legends, and the vertical lines are the standard uncertainties.

**Fig. 5.6:**
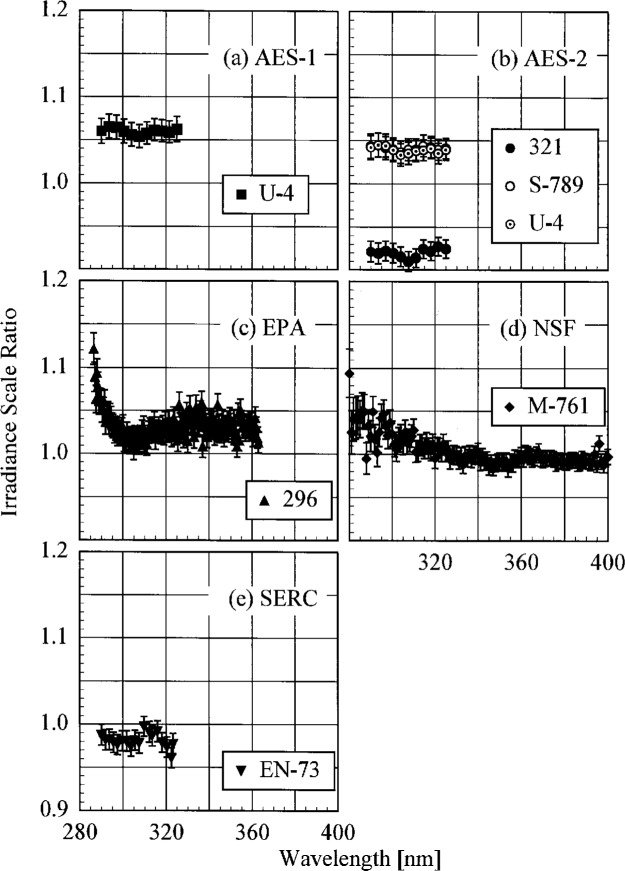
Ratio of the participants’ spectral irradiance scales to the NIST spectral irradiance scale as a function of wavelength from spectral scans performed outdoors. The instruments are indicated in each panel, the participants’ lamps are indicated in the legends, and the vertical lines are the standard uncertainties.

**Fig. 5.7:**
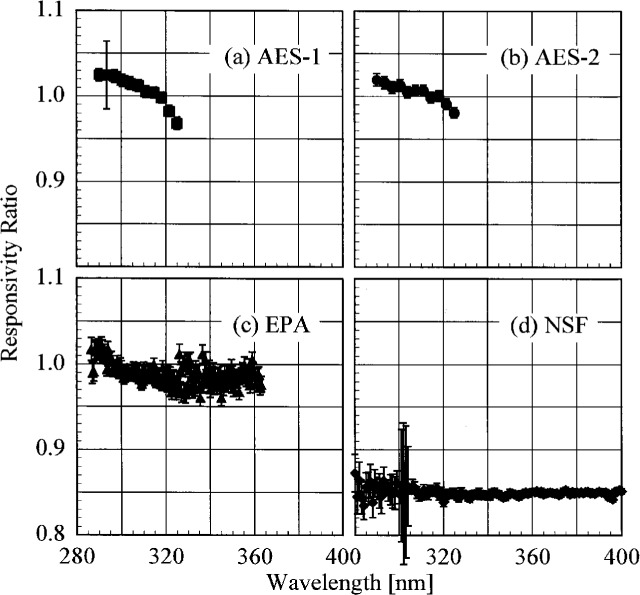
Ratio of the responsivity determined outdoors to the responsivity determined indoors, using the NIST standard lamp, as a function of wavelength, indicating the translational stability of the instruments. The instruments are indicated in each panel, and the vertical lines are the standard uncertainties.

The participant responsivities used for this comparison were those that were determined on the same day as a scan of the NIST standard lamp. The ratio of the participant’s spectral irradiance scale to the NIST scale as a function of wavelength is shown in Fig. 5.5 for scans performed indoors and in Fig 5.6 for scans performed outdoors. The instruments are indicated in each panel, while the participants’ lamps are indicated in the legends. The lamps, times, and instrument temperature changes used for the ratios in these two figures, as well as in Figs. 5.7 to 5.10, are listed in Table 5.4. The spectral irradiance scales from the DXW lamps used by AES are consistently greater than the NIST irradiance scale by less than 5 % both indoors and outdoors, except for the one determined outdoors with lamp U-4 for AES-1, shown in Fig. 5.6(a). As discussed below, the responsivity of the Brewer instruments depends on temperature, and the increase of the scale ratio shown in Fig. 5.6(a) is consistent with the temperature change between the spectral scans, given in Table 5.4. The disagreement is greater for the irradiance scale from the Sci-Tec lamps used by both AES and EPA. The irradiance scale from the lamps used by AES was consistently lower than the NIST irradiance scale by less than 9 %, whereas the irradiance scale from the lamps used by EPA was consistently higher than the NIST irradiance scale by less than 10 %, except at the shortest wavelengths, where it was higher by as much as 15 %. The irradiance scale used by NSF was consistently greater than the NIST irradiance scale by less than 5 % for wavelengths shorter than approximately 330 nm, while it was consistently less by less than 2 % for longer wavelengths. The irradiance scale used by SERC was consistently lower than the NIST irradiance scale by less than 4 %. This comparison of the participants’ spectral irradiance scales indicates agreement within 10 % of the NIST irradiance scale.

The responsivity of every instrument except that from SERC was determined both indoors and outdoors. Therefore, the ratio of the responsivity determined outdoors with a particular lamp to the responsivity determined indoors with the same lamp indicates the stability of the instrument to movement, termed translational stability.

**Fig. 5.8:**
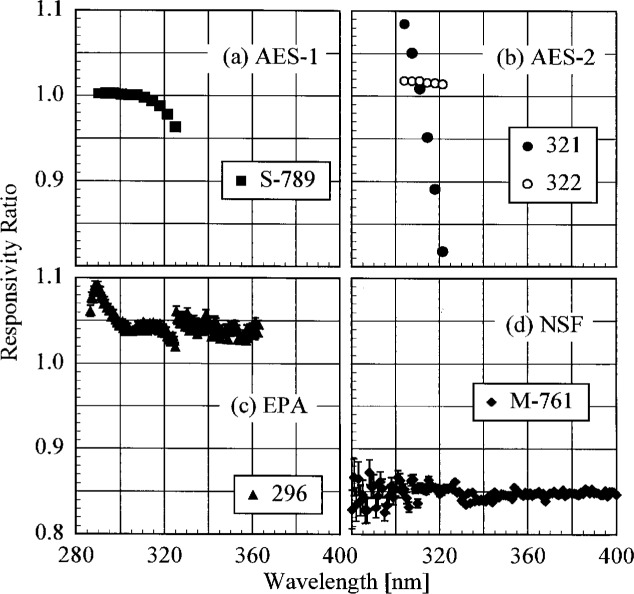
Ratio of the responsivity determined outdoors to the responsivity determined indoors, using the participants’ lamps, as a function of wavelength, indicating the translational stability of the instruments. The instruments are indicated in each panel, and the vertical lines are the standard uncertainties.

The translational stability was determined using both the NIST standard lamp and participants’ lamps. The first outdoor determination of responsivity with a lamp that had also been scanned indoors was used for the ratio. The ratio of the responsivity determined outdoors to that determined indoors as a function of wavelength is shown in Fig. 5.7 from spectral scans using the NIST standard lamp and in Fig. 5.8 from spectral scans using the participants’ lamps. The instruments are indicated in each panel, while the participants’ lamps are indicated in the legends. The two AES instruments were stable to within 3 % and 2 %, respectively, as determined by the NIST standard lamp, with a noticeable wavelength dependence. This stability and wavelength dependence was consistent with that obtained with the participants’ lamps, except with lamp 321, which had an anomalously severe wavelength dependence. The EPA instrument had a consistently lower responsivity outdoors by less than 4 % for wavelengths longer than 325 nm, while there is some wavelength dependence for shorter wavelengths. The participant’s lamp indicated less stability, within 5 % at most wavelengths and with a pronounced wavelength dependence at wavelengths shorter than 325 nm. The NSF instrument suffered a relatively large reduction in responsivity of approximately 15 % upon moving outdoors, which is obviously a property of the instrument since the same reduction was observed with both the NIST and the NSF lamps.

The responsivity of every instrument was determined twice outdoors using the NIST standard lamp, and most participants also determined responsivities based upon their lamps several times outdoors. The ratio of responsivities determined with the same lamp at two different times indicates the temporal stability of the instrument.

**Fig. 5.9:**
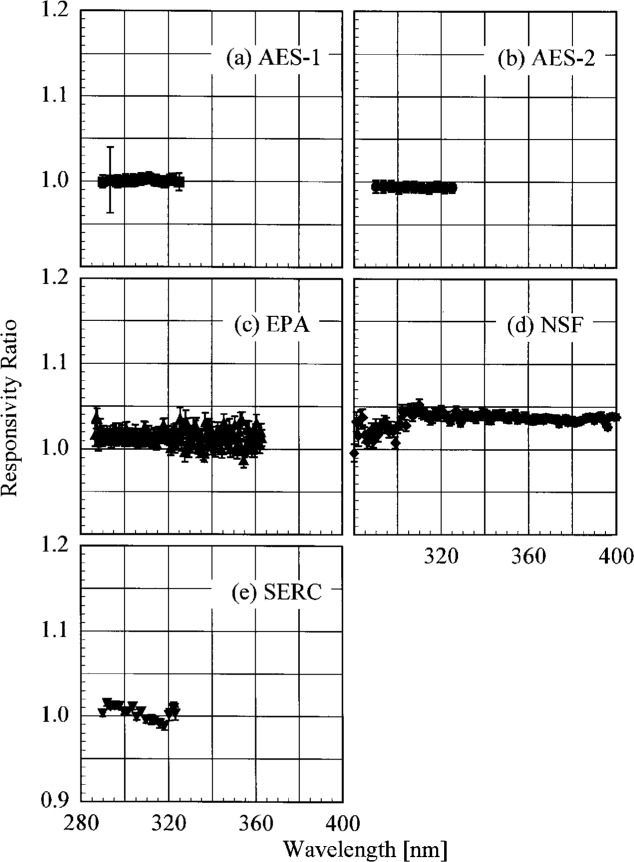
Ratio of two responsivities determined outdoors, using the NIST standard lamp, as a function of wavelength, indicating the temporal stability of the instruments. The instruments are indicated in each panel, and the vertical lines are the standard uncertainties.

**Fig. 5.10:**
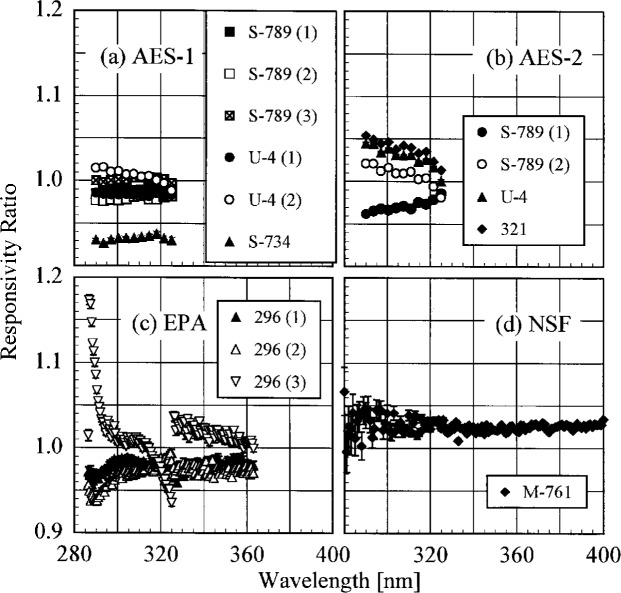
Ratio of two responsivities determined outdoors, using the participants’ lamps, as a function of wavelength, indicating the temporal stability of the instruments. The instruments are indicated in each panel, and the vertical lines are the standard uncertainties. For multiple ratios with the same lamp, the time order of the ratios is also indicated in the legend.

The ratio of the responsivity determined outdoors to a previous determination with the same lamp outdoors as a function of wavelength is shown in Fig. 5.9 from spectral scans using the NIST standard lamp and in Fig. 5.10 from spectral scans using the participants’ lamps. The instruments are indicated in each panel, while the participants’ lamps are indicated in the legends. The results with the NIST standard lamp indicate that the instruments are stable to within 5 % over several days with no wavelength dependence. In the best case, the AES-1 instrument was stable to within 0.5 %. However, the effects of temperature discussed below indicate that this agreement is fortuitous. The temporal stability determined by the participants’ lamps is less impressive. The stability of the AES instruments was within 5 %, with a slight wavelength dependence, except for that determined with lamp S-734. The stability of the EPA instrument is also generally within 5 %, but there is a marked wavelength dependence at wavelengths shorter than 325 nm, and the responsivity ratio increases to 17 % at the shortest wavelength for one ratio. The stability of the NSF instrument obtained with the participant’s standard lamp corroborates that obtained with the NIST standard lamp.

A complication in interpreting the results shown in Figs. 5.7 to 5.10 for the Brewer instruments are temperature and wavelength registration effects. Specifically, the smooth wavelength dependence of the responsivity ratios shown in Figs. 5.7(a), 5.7(b), 5.8(a), 5.8(b), 5.10(a) (for the second ratio using lamp U-4), 5.10(b), and 5.10(c) (for the first and second ratios) are all likely due to the temperature of the instrument. While there is no temperature control of either the instrument or the PMT, the internal temperature is monitored close to the NiSO_4_ filter in front of the PMT. It is known that the transmittance of this filter is inversely proportional to the temperature, and has a wavelength dependence, and indeed the calculation of total column ozone accounts for this temperature and wavelength dependence. However, no temperature correction is applied to spectral scans, and so the responsivity should decrease as the temperature increases, with a wavelength dependence.

The changes in responsivity shown in the figures mentioned in the preceding paragraph are consistent with the temperature changes given in Table 5.4. With only a few exceptions, an increase in the temperature of a Brewer instrument between two spectral scans used in a ratio resulted in a decrease in responsivity, while the opposite effect occurred with a decrease in temperature. The large ratio for lamp 321 in Fig. 5.8(b) is unexplained. Exceptions to the general rule of an increase in temperature leading to a decrease in responsivity all occurred for the EPA instrument, as shown in Figs. 5.7(c) and 5.9(c) and Table 5.4. The excellent temporal stability demonstrated by the two AES instruments in Fig. 5.9 is likely due to the small temperature changes of the instruments between the spectral scans.

**Fig. 5.11:**
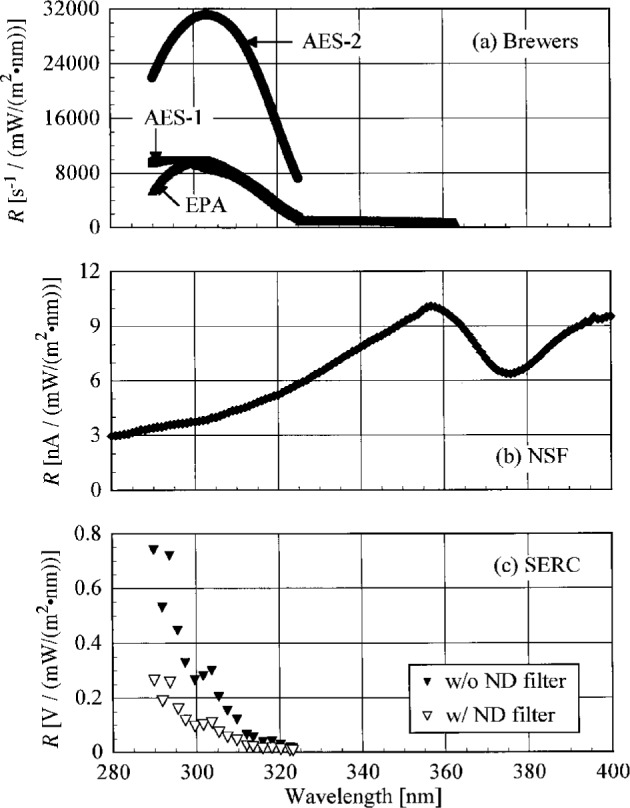
Responsivity *R* as a function of wavelength for each instrument, as indicated in the panels. The responsivity of the EPA instrument extends to 363 nm, and the responsivity of the SERC instrument was determined both without andwith a neutral-density filter over the diffuser, as indicated inthe legend.

The wavelength dependence, at wavelengths shorter than 325 nm, of the responsivity ratios shown in Figs. 5.8(c) and 5.10(c) (for the third ratio) is most likely the result of a shift in the wavelength registration of the EPA instrument. The responsivity of this instrument is shown in Fig. 5.11(a), and is peaked at 319 nm for wavelengths shorter than 325 nm (because of the NiSO_4_ filter), and is relatively flat for longer wavelengths. The derivative of this responsivity with respect to wavelength has the same shape as the responsivity ratios in Figs. 5.8(c) and 5.10(c), and indicates that wavelength registration shifts by approximately 0.2 nm and 0.5 nm, respectively, account for the magnitudes shown in these figures. This could happen if the wavelength registration of the instrument was not set prior to one of the spectral scans used for the ratios.

Several important conclusions can be drawn from the determinations of responsivity. Those made with the NIST standard lamp show that the responsivity changes upon movement of the instruments, and was especially pronounced for the NSF instrument. The effect of temperature on the Brewer instruments obscures their translational stability. Therefore, the responsivity of an instrument must be determined at the site where the instrument will be used for monitoring, under the same conditions that are expected at that site. It is not sufficient to determine the responsivity off-site and then expect the instrument to have the same responsivity when it is moved to its monitoring location. The results also show that the responsivities have temporal stability generally within 5 %, at least over the several days of the Intercomparison, when the temperature was approximately the same during the two determinations. More generally, it was quite advantageous to use a common standard lamp that was used carefully and consistently for all the instruments at the Intercomparison. This allowed determinations of the translational and temporal stability of the instruments without the added complications of differing lamps and techniques between instruments and participants. Using a common standard for responsivity also simplifies the intercomparison between instruments since differences between spectral irradiance scales are removed and the actual instrument performances can be evaluated more readily.

The use of participants’ lamps to determine instrument responsivity was more problematic. The agreement of the participant spectral irradiance scales to within only 10 % of the NIST scale greatly complicates comparisons of solar irradiance measured by different instruments, especially those from different networks. There is thus a need for the participants to critically analyze their irradiance scale transfer chains to improve the agreement between their scales and the one maintained by NIST. An investigation of the irradiances of calibrated standard lamps issued by different manufacturers would be useful in such an analysis. This large spread in the scales also illustrates the need to use a set of common standard lamps within a network and between networks so that the irradiance scales for all the instruments are the same.

The responsivities determined from the first outdoor scans of the NIST standard lamp were used for the synchronized solar scans. The responsivities were interpolated with a natural cubic-spline to the wavelengths of the synchronized scans. The responsivity of each instrument as a function of wavelength is shown in Fig. 5.11 for (a) the Brewer instruments, (b) the NSF instrument, and (c) the SERC instrument. The standard uncertainties are the sizes of the symbols and thus are not shown.

The agreement of the participants’ spectral irradiance scales with the NIST scale is similar to results from other Intercomparisons. The first European Intercomparison showed agreement between the scales of the participating instruments of only 20 %, which was improved to 5 % for the second and third Intercomparisons [2–4]. A complication in these Intercomparisons was the orientation of the standard lamps. These lamps were operated in a vertical position, meaning that the instruments had to be turned on their side to view the lamps. This was avoided at the North American Intercomparison by using standard lamps operating in the horizontal position. Likewise, the Finnish Intercomparison used a horizontal standard lamp and achieved agreement between the scales of the participating instruments of 10 % [5].

The uncertainty values shown in Tables 5.2 and 5.3 indicate that the component that contributes the greatest uncertainty is the lamp irradiance. The well-designed power supply for the lamp current is reflected in the small relative standard uncertainties from this component. The major component of uncertainty from alignment of the lamp is the distance between the jig and the diffuser, illustrating the care with which this distance needs to be determined.

## 6. Solar Irradiance

### 6.1 Introduction

The ultimate goal of the Intercomparison was to have all the instruments measure the solar ultraviolet irradiance concurrently. This was achieved over several days of the Intercomparison. The solar ultraviolet irradiance *E*(*λ*_0_) was calculated from the measured signals *S*(*λ*_0_) using the simplified measurement equation given by

$$E\left(\lambda_{0}\right)=S\left(\lambda_{0}\right)/R\left(\lambda_{0}\right),$$ (6.1)

with the responsivity *R*(*λ*_0_) for each instrument being that determined from the first outdoor scan of the NIST standard lamp. This was done to provide a common irradiance scale for all the instruments, thereby facilitating comparisons between instruments.

### 6.2 Experimental Procedure

Synchronized spectral scans of the solar irradiance began on the hour and half-hour from wavelengths of 290 nm to 325 nm at increments of 0.25 nm with 3 s between each wavelength. This range was common to all the instruments, and the EPA and NSF instruments continued scanning to longer wavelengths. The clock for each instrument was set daily from a common clock synchronized with the satellite Global Positioning System. The synchronized scans lasted 7 min, and the maximum discrepancy in time between instruments during these scans was 2 s. Other measurements, such as wavelength calibrations and total column ozone, were performed by some instruments during the time between synchronized scans. The days, times, and participating instruments for the synchronized solar scans used in the analyses below are listed in Table 6.1. All synchronized scans occurred after the snow had melted. Calibrations of the instruments reduced the number of synchronized scans in which all the instruments participated.

### 6.3 Data Analysis

To keep the signal from the SERC instrument within the linear range of the detector, a quartz neutral density filter was placed over the diffuser during the middle of the day. The filter transmittance at each wavelength channel was calculated from the signals obtained from solar scans with and without the filter. Using the responsivity of the instrument determined without the filter, the solar irradiance for each channel was calculated for approximately 30 min before and after the filter was either placed over or removed from the diffuser. Linear fits of irradiance, both with and without the filter, as a function of time were extrapolated to the time when the filter was changed. The ratio of the extrapolated irradiance with the filter to that without the filter is the filter transmittance. The transmittances at each channel from seven repetitions of the filter changes were averaged to obtain the filter transmittance as a function of wavelength, with the standard deviation of the mean as the standard uncertainty. A linear fit of the filter transmittance as a function of wavelength for wavelengths longer than 300 nm was used to obtain the transmittances for shorter wavelengths. The transmittance was approximately 0.36 and increased slightly with increasing wavelength. For those solar scans during which the neutral density filter was over the diffuser, the responsivity determined from the NIST standard lamp was multiplied by the filter transmittance to obtain the responsivity with the filter. This responsivity is shown in Fig. 5.11(c).

The method used to determine the responsivity of the NSF instrument during solar scans complicated the data analysis. The usual procedure with this instrument is to transfer the spectral irradiance scale of the external 200 W lamp to the internal 45 W lamp from spectral scans of both lamps with the same high voltage on the PMT. Different high voltages are used for scans of the solar irradiance, and the responsivity of the instrument is dependent upon the high voltage. Therefore, the internal lamp is scanned at least daily at these high voltages to determine the responsivity under these conditions. To use the NIST irradiance scale with this procedure, the scale was transferred to the NSF external 200 W lamp from the first outdoor scan of the NIST standard lamp, and this new scale for the external lamp was then used with scans of the internal 45 W lamp. The responsivity of the instrument at any high voltage was determined from the scan of the 45 W lamp at the same high voltage that occurred closest in time to the scan of the solar irradiance.

For all instruments, the measured signal was corrected before the irradiance was calculated. For the Brewer instruments, the signal was converted to a photon rate as detailed in Sec. 3.1 with dark subtraction and dead-time correction. The wavelengths of the NSF instrument were corrected as detailed in Sec. 3.2, while dark subtraction was performed by averaging all the signals at wavelengths shorter than 290 nm and subtracting this value from all the signals of the scan. Dark subtraction and averaging the signals over the 7 min of the synchronized scans was performed for the SERC instrument.

As shown in Fig. 5.2, the stray-light rejection of the instruments can result in relatively large signals at the shortest wavelengths. To account for this, stray-light subtraction was employed for the Brewer instruments. The signals at wavelengths shorter than 292.75 nm were averaged and subtracted from all signals from the scan. It was these signals with the stray-light subtraction that were divided by the responsivity to obtain the solar ultraviolet irradiance. The subtraction used for the NSF instrument is also a stray-light subtraction, although the signals obtained in a darkened room with no source illuminating the diffuser are the same as those obtained with the solar spectral scans at wavelengths shorter than 290 nm. Therefore, the contribution to the signal due to stray-light is indistinguishable from the dark signal.

### 6.4 Results and Discussion

#### 6.4.1 Introduction

**Fig. 6.1:**
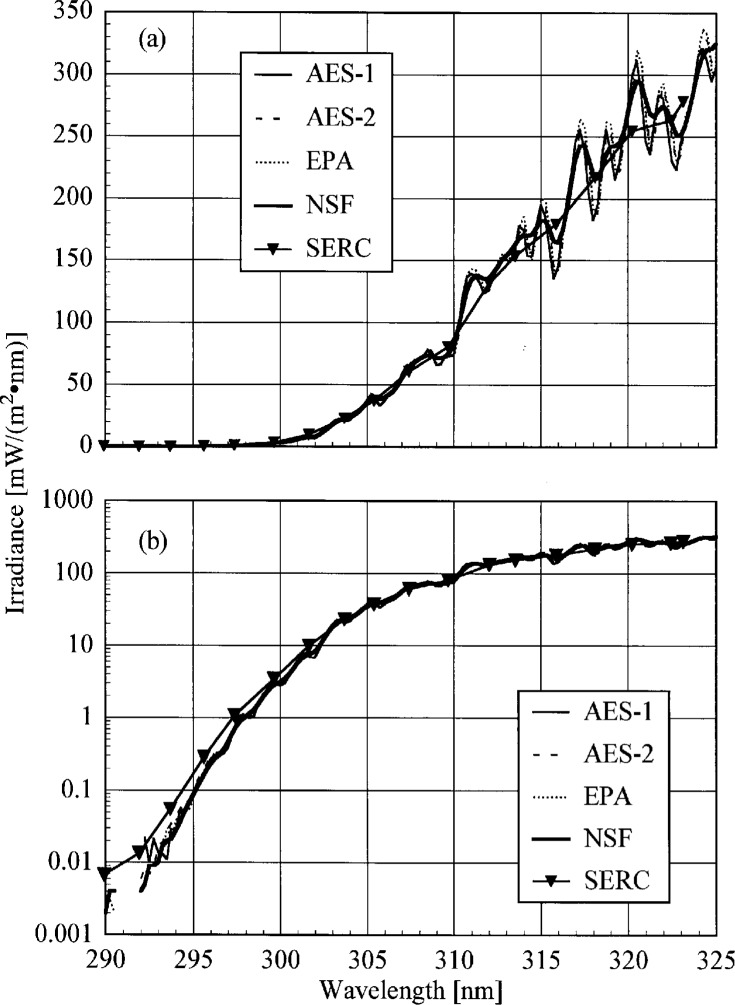
Solar irradiance on an (a) linear and (b) logarithmic scale as a function of wavelength determined by the instruments indicated in the legend on day 267 at 19:00 h.

The solar irradiance as a function of wavelength determined by all instruments from a synchronized spectral scan on day 267 at 19:00 h is shown in Fig. 6.1. The irradiance is plotted on a linear scale in Fig. 6.1(a) and on a logarithmic scale in Fig. 6.1(b). This figure illustrates the challenges encountered in accurately measuring the solar ultraviolet irradiance, especially in the UV-B wavelength region, and of comparing the results between instruments. The outstanding feature of ground-level solar ultraviolet irradiance is its rapid decrease with decreasing wavelength in the UV-B region due to absorption by ozone, as illustrated in Fig. 6.1(b). The irradiance decreases by nearly five orders of magnitude from 325 nm to 290 nm, which imposes stringent requirements on the instruments in terms of wavelength accuracy and stray-light rejection. In the region of steepest decrease, a relatively small uncertainty in wavelength translates into a relatively large uncertainty in irradiance. An accurate measurement of the irradiance at the shortest wavelengths requires the best possible stray-light rejection so the signal is not dominated by light from wavelengths longer than the nominal one.

The moderately structured nature of the solar spectral irradiance, as shown in Fig. 6.1(a) for wavelengths greater than 310 nm, complicates comparisons between instruments. While the structure of the spectral irradiance is consistent among instruments, with maxima and minima occurring at the same wavelengths, the effect of the different bandwidths is also apparent. As the bandwidths of the instruments increases, from the Brewers to NSF to SERC, the measured spectral irradiance becomes smoother. The maxima and minima measured by the NSF instrument are not as pronounced as they are with the Brewer instruments, and virtually no structure is evident with the SERC instrument. The effect of the wider bandwidth of the SERC instrument, combined with the rapid decrease in solar irradiance, is also apparent in Fig. 6.1(b). The irradiance measured by the SERC instrument is greater than that measured by the other instruments at wavelengths shorter than 305 nm because the signal from each filter channel is predominately weighted by the irradiance at wavelengths greater than the center wavelength of that filter. One method for taking this effect into account is to use an effective center wavelength for each filter [11], which brings the irradiance measured by the SERC instrument more into agreement with that measured by the other instruments [16]. However, that approach requires an estimate of the actual solar irradiance, so it will not be discussed further in this paper.

The problem remains of how to compare the solar irradiances measured by instruments with different bandwidths. While deconvolution and spectral synthesis techniques are being investigated, the approach taken for this paper, which is conceptually the simplest, is to convolve the irradiances with a common slit-scattering function. This assumes that the instruments are accurately measuring the solar irradiance, so that the convolution is the solar irradiance that would be obtained by a hypothetical instrument with a given slit-scattering function. The results are presented in order of increasing bandwidth for the slit-scattering function used in the covolution. First, the solar irradiances from the three Brewer instruments are compared, since they comprised the majority of the instruments at the Intercomparison and they all had the same nominal bandwidth so no convolution is necessary. Next, the solar irradiances from the scanning spectroradiometers are compared, the three Brewers and the NSF instrument, by convolving each irradiance with a 1 nm FWHM ideal triangular slit-scattering function. Finally, the solar irradiances from all the instruments are compared by convolving the irradiances from the scanning instruments with the filter transmittances of the SERC instrument. The value used to quantify the agreement between instruments is the standard deviation of the solar irradiances divided by the average irradiance at each wavelength, expressed as the relative standard uncertainty.

**Fig. 6.2:**
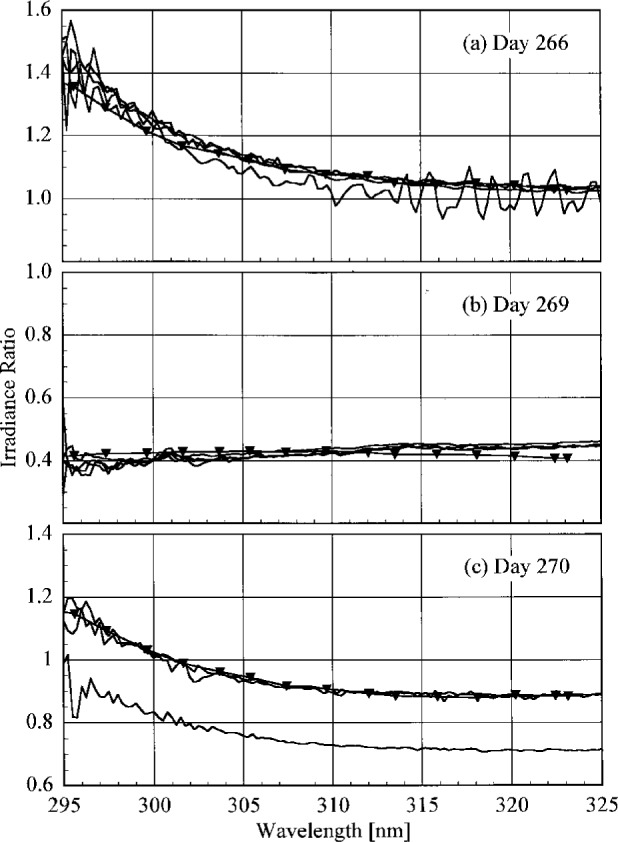
Ratio of solar irradiance as a function of wavelength determined on the day indicated in the panel to that determined on day 267, both at 19:00 h, for each instrument. The symbols are for the SERC instrument, the curve with the large fluctuations in (a) is for the AES-1 instrument, and the curve below the others in (c) is for the EPA instrument.

Since the goal of all the monitoring networks is to detect changes in solar ultraviolet irradiance due to ozone depletion, it is instructive to compare the irradiances measured by each instrument on different days. This also serves to determine if an instrument is not operating properly on one of the days. Since the best atmospheric conditions and results occurred on day 267, the solar irradiances measured on other days at 19:00 h were divided by those measured on day 267 at 19:00 h. The results are shown in Fig. 6.2, where the ratio of the irradiances is plotted as a function of wavelength for the days indicated in the panels. The symbols are for the SERC instrument.

The first result of note is that the solar irradiances from the same instrument can be reasonably compared at wavelengths as short as 295 nm, which is 5 nm shorter than was achievable when comparing among instruments. The curve with the much more pronounced spectral structure in Fig. 6.2(a) is that of the AES-1 instrument. This implies a problem with this instrument on day 266, and so the solar irradiances measured by it on this day are not included in the following analyses. The lower curve in Fig. 6.2(c) is that of the EPA instrument, which resulted from the diffuser being moved on the previous evening. Finally, the results obtained with the SERC instrument agree very well with those obtained with the scanning instruments, and the absence of any noticeable spectral structure in the curves indicates that the instruments had good wavelength stability.

The results can be understood from the atmospheric conditions at the times of the measurements. The aerosol optical depths shown in Fig. 4.4, as well as the irradiances from the pyranometers and pyrheliometer, indicate that the sky was progressively more turbid over the course of the Intercomparison. Obviously, there were clouds on day 269 at 19:00 h, as shown in Fig. 4.3(d). The total column ozone, however, increased from 295 Pa·m (291 matm·cm) on day 266 to 307 Pa·m (303 matm·cm) on day 267, then decreased to 286 Pa·m (282 matm·cm) on day 270. The solar irradiance was greater on day 266 than on day 267 because the sky was less turbid and the total column ozone was lower. The turbidity was responsible for the ratio being greater than one, and relatively constant with wavelength, for wavelengths longer than 310 nm, while the rapid increase in the ratio with decreasing wavelength at wavelengths shorter than 310 nm was due to the total column ozone. Conversely, the solar irradiance was less on day 270 than on day 267 for wavelengths longer than 310 nm, again relatively constant with wavelength, because the sky was more turbid, while at shorter wavelengths the ratio increased because the total column ozone was lower. These results indicate that all the instruments are capable of detecting changes in solar irradiance due to total column ozone, although the effects on the absolute solar irradiance are complicated by the turbidity of the sky.

#### 6.4.2 Brewer Instruments

**Fig. 6.3:**
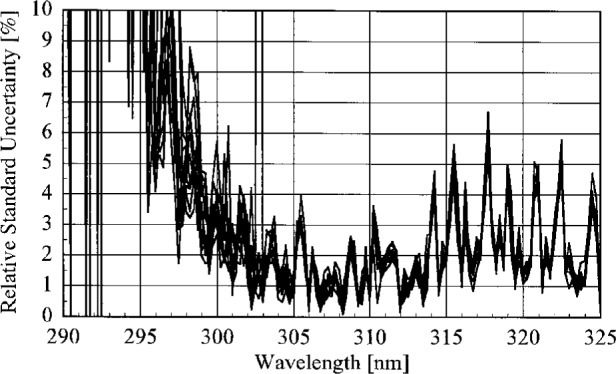
Relative standard uncertainty as a function of wavelength of the solar irradiances determined by the three Brewer instruments AES-1, AES-2, and EPA for all eleven synchronized solar scans on day 267.

The relative standard uncertainty of the solar irradiances measured by the three Brewer instruments is shown in Fig. 6.3 for all eleven synchronized scans performed on day 267. The relative standard uncertainties are wildly variable for wavelengths shorter than 300 nm, while they are remarkably consistent between synchronized scans for longer wavelengths. The spectral structure of the relative standard uncertainties, especially its consistency between scans, implies a correlation with the spectral structure of the solar irradiance. Given that the instruments are nominally the same, their responsivities were determined using the same standard lamp, and they are measuring under identical conditions, ideally the solar irradiances should be equal and the relative standard uncertainty would then be zero at all wavelengths. However, the relative standard uncertainty has spectral structure and can be larger than 6 %.

To determine the various effects that are responsible for the relative standard uncertainties shown in Fig. 6.3, the analysis will concentrate on the results obtained on day 267 at 19:00 h. As shown in Figs. 4.3(b) and 4.4, the atmosphere was cleanest and clearest on this day of the Intercomparison, and since 19:00 h is the time closest to solar noon, the irradiance is changing the least with time. To quantify the correlation of the spectral structure of the relative standard uncertainty with that from the solar irradiance, other contributions to the relative standard uncertainty are determined first.

Some of the relative standard uncertainty arises from random effects: the signals from solar and standard lamp spectral scans, and the alignment of the standard lamp. Propagating the relative standard uncertainties of the signals from the solar spectral scans yields a combined relative standard uncertainty of 8 % at 290 nm which decreases to 0.4 % at 305 nm and remains constant at longer wavelengths. Therefore, the component to the relative standard uncertainty arising from the signals from the solar spectral scans is only 0.4 %. The relative standard uncertainty in the responsivity from the signals and standard lamp alignment are 0.5 % for each instrument, which combine to be 0.9 % for all three. The root-sum-square of the relative standard uncertainties arising from random effects during the solar and standard lamp spectral scans is thus only 1 %.

As shown in Fig. 5.9, the responsivity of each instrument changes over time. The average of the responsivity ratios shown in Fig. 5.9 for wavelengths shorter than 325 nm are 0.1 % for AES-1, 0.6 % for AES-2, and 1.5 % for EPA. The root-sum-square of these values is 1.6 %. Combining the relative standard uncertainties arising from random effects and from changes in responsivity yields a combined relative standard uncertainty of 2 %. Since this value is generally lower than the relative standard uncertainties shown in Fig. 6.3, and is constant as a function of wavelength, correlations of the spectral structure of the relative standard uncertainty with that of the solar irradiance are discussed next.

**Fig. 6.4:**
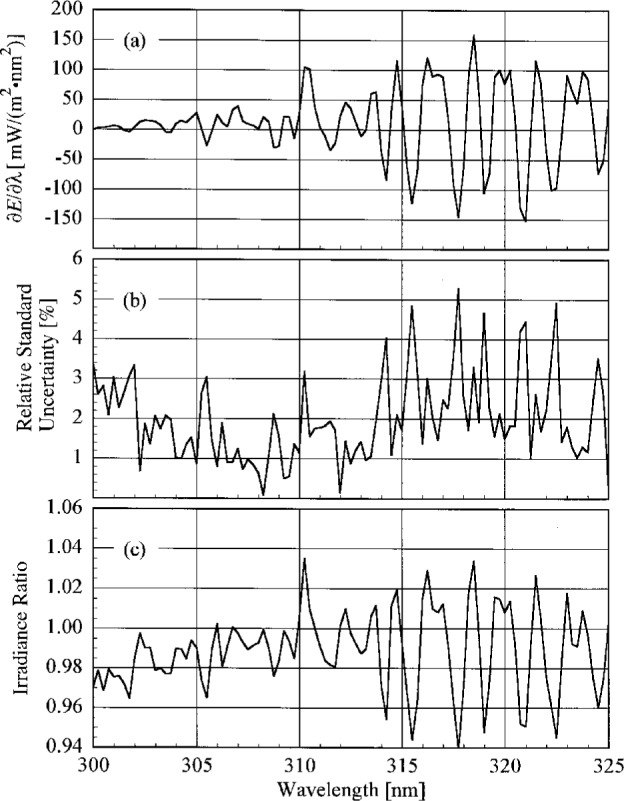
(a) Derivative of the average solar irradiance with respect to wavelength, (b) relative standard uncertainty, and (c) ratio of solar irradiance determined by AES-1 to the average solar irradiance as a function of wavelength for the solar irradiances determined by the three Brewer instruments AES-1, AES-2, and EPA on day 267 at 19:00 h.

The derivative of the average solar irradiance with respect to wavelength, obtained with a natural cubic spline interpolation, as a function of wavelength is shown in Fig. 6.4(a). The relative standard uncertainty as a function of wavelength is shown in Fig. 6.4(b), and the ratio of the solar irradiance determined by AES-1 divided by the average solar irradiance as a function of wavelength is shown in Fig. 6.4(c). All of these are from measurements at 19:00 h on day 267. There is a definite correlation between the derivative of the average irradiance and the relative standard uncertainty: at wavelengths where the magnitude of the derivative is large the relative standard uncertainty is also large, and where the derivative is small the relative standard uncertainty is also small. This suggests that there is a wavelength uncertainty among the Brewer instruments. The relative standard uncertainty of irradiance *u*(*E*)/*E*(*λ*) in terms of a wavelength standard uncertainty *u*(*λ*) is given by

$$\frac{u\left(E\right)}{E\left(\lambda \right)}=\left(\frac{\partial E\left(\lambda \right)}{\partial \lambda }\right)\left(\frac{u\left(\lambda \right)}{E\left(\lambda \right)}\right).$$ (6.2)

Knowing *u*(*E*)/*E* (*λ*), ∂*E* (*λ*)/∂*λ*, and *E* (*λ*), the wavelength standard uncertainty *u*(*λ*) can be calculated. Removing the 2 % relative standard uncertainty from the components detailed above, performing the calculation for *u*(*λ*), and averaging the values at those wavelengths at which *u*(*λ*)was greater than zero and |*E* (*λ*)/*λ* | was greater than 10, yields *u* (*λ*) = 0.06 nm. This value for the standard uncertainty of the wavelength is consistent with the wavelength standard uncertainties detailed in Sec. 5.2. Therefore, a wavelength uncertainty of 0.06 nm among the three Brewer instruments, which is consistent with the wavelength uncertainties determined by other techniques, is responsible for the magnitude and spectral structure of the relative standard uncertainty of the solar irradiance.

This conclusion is further strengthened by the ratio of the solar irradiance measured by the AES-1 instrument to the average solar irradiance, shown in Fig. 6.4(c). If the wavelength of one instrument is shifted, relative to the average wavelength, to shorter (longer) wavelengths, then the ratio of the solar irradiance measured by that instrument to the average will be greater than unity (less than unity) when the average irradiance is increasing (decreasing). This is precisely what occurs when com paring Figs. 6.4(a) and 6.4(c). The average solar irradiance is increasing when its derivative is greater than zero, and the irradiance ratio is also greater than unity, while the ratio is less than unity when the derivative is less than zero. Therefore, the wavelength of the AES-1 instrument was shifted to shorter wavelengths relative to the average. The ratios of the solar irradiances of the other instruments to the average show that their wavelengths were shifted to longer wavelengths, with AES-2 closest to the average. Thus, the spectral structure of the relative standard uncertainty of the solar irradiances of the three Brewer instruments is consistent with a wavelength uncertainty among them.

The relative standard uncertainty as a function of wavelength of the three Brewer instruments on day 269 at 19:00 h is almost identical to that shown in Fig. 6.4(b). This agreement is fairly remarkable given that the sky was cloudy on day 269, as shown in Fig. 4.3(d), and indicates the consistency that can be achieved between similar instruments.

**Fig. 6.5:**
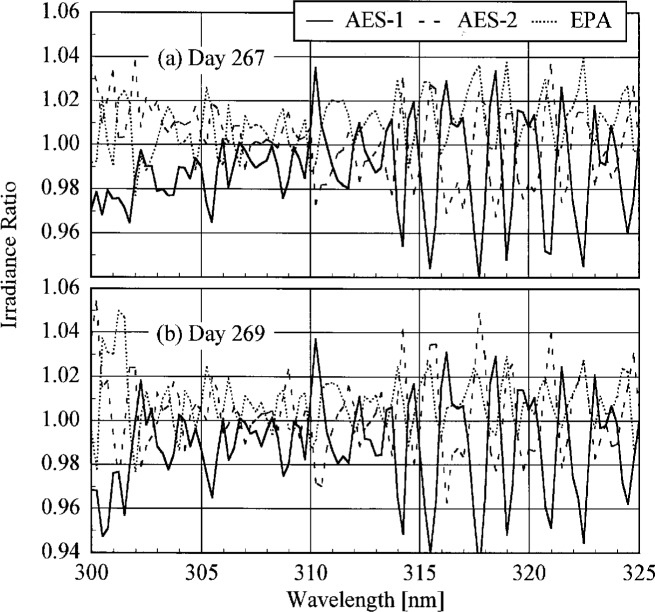
Ratio of solar irradiance measured by each instrument to the average irradiance as a function of wavelength by the three Brewer instruments AES-1, AES-2, and EPA on the days indicated in the panels at 19:00 h.

Other intercomparisons report the agreement between the solar irradiances measured by different instruments as ratios of the measured irradiances to the irradiance of one particular instrument that was chosen as a reference. The designation of a reference instrument was not appropriate for this Intercomparison, so instead the relative standard uncertainty of the irradiances is used. This is equivalent to the ratios of irradiances measured by individual instruments to a reference irradiance. Since all the instruments performed synchronized spectral scans under nominally identical conditions, they are all assumed to have been exposed to the same spectral irradiance. However, each instrument measured an independent value for the irradiance, and therefore the relative standard uncertainty of these independent values is used to indicate the agreement between instruments. To make a connection with the results presented for other intercomparisons, and to corroborate the use of relative standard uncertainties to describe the agreement between instruments, the ratios of the irradiances measured by the three Brewer instruments to the average irradiance as a function of wavelength at 19:00 h are shown in Fig. 6.5 on the days indicated. The ratios are bounded by 0.95 and 1.05 at all but a few wavelengths, which agrees very well with the relative standard uncertainties shown in Fig. 6.4(b).

**Fig. 6.6:**
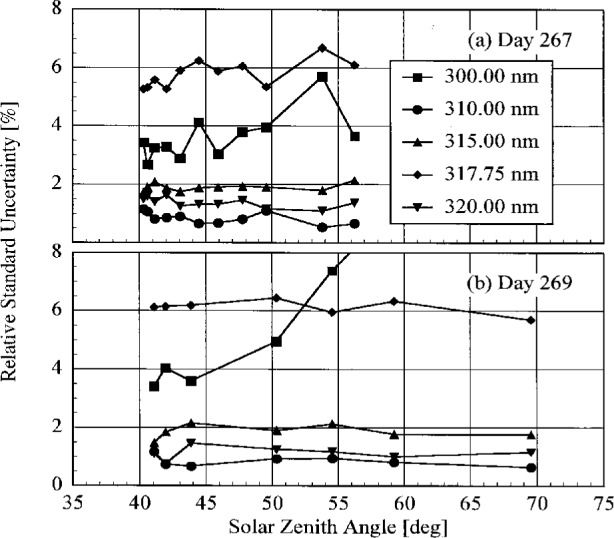
Relative standard uncertainty of the solar irradiances as a function of solar zenith angle at the wavelengths indicated in the legend determined by the three Brewer instruments AES-1, AES-2, and EPA on the days indicated in the panels.

The relative standard uncertainty of the solar irradiances as a function of solar zenith angle is shown in Fig. 6.6 on the days indicated in the panels at the wavelengths indicated in the legend. The wavelengths were chosen to be representative of the spectral structure of the relative standard uncertainty, especially 317.75 nm since this is the wavelength with the maximum relative standard uncertainty. The relative standard uncertainty at 300 nm increases with increasing solar zenith angle since the irradiance at this wavelength is relatively small, resulting in a lower signal-to-noise ratio and hence a larger relative standard uncertainty. The relative standard uncertainties at the other wavelengths are approximately constant as a function of solar zenith angle, except for the ones at 317.75 nm on day 267, as expected for instruments that are nominally similar.

#### 6.4.3 Scanning Instruments

**Fig. 6.7:**
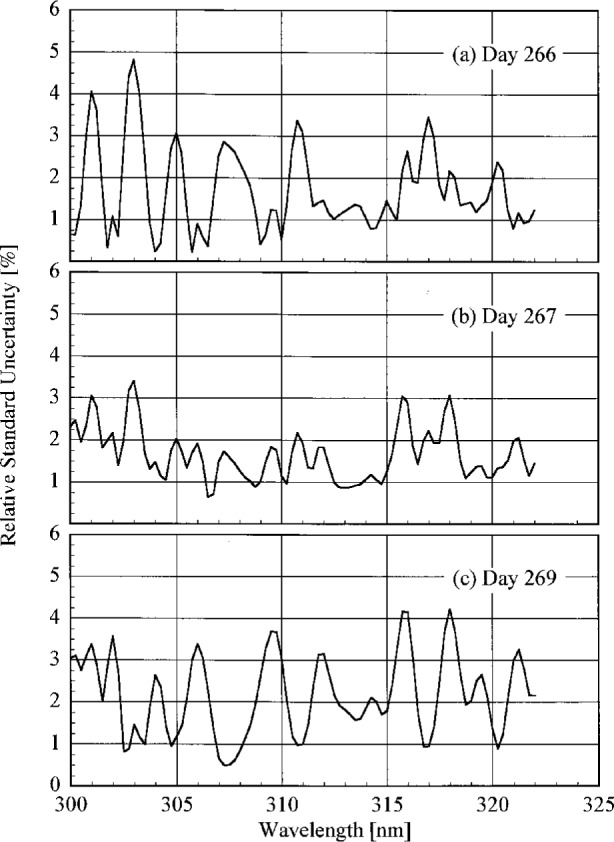
Relative standard uncertainty of the solar irradiances as a funciton of wavelength determined by the four scanning instruments AES-1, AES-2, EPA, and NSF on the days indicated in the panels at 19:00 h. The solar irradiances determined by each instrument were convolved with a 1 nm FWHM ideal triangular slit-scattering function.

**Fig. 6.8:**
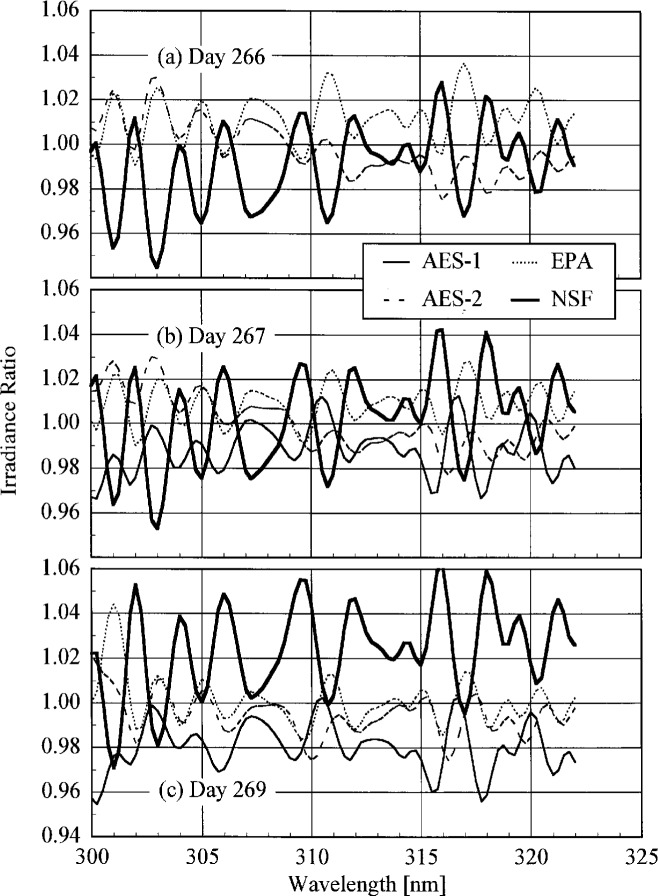
Ratio of solar irradiance measured by each instrument to the average irradiance as a function of wavelength by the four scanning instruments AES-1, AES-2, EPA, and NSF on the days indicated in the panels at 19:00 h. The solar irradiances determined by each instrument were convolved with a 1 nm FWHM ideal triangular slit-scattering function.

To compare the solar irradiances measured by the four scanning instruments, AES-1, AES-2, EPA, and NSF, the irradiances were all convolved with a 1 nm FWHM ideal triangular slit-scattering function. The relative standard uncertainty of the solar irradiances as a function of wavelength measured at 19:00 h is shown in Fig. 6.7 on the days indicated in the panels. The ratios of the irradiances measured by each instrument to the average irradiance as a function of wavelength at 19:00 h is shown in Fig. 6.8 on the days indicated in the panels. Again, there is excellent agreement between the relative standard uncertainties shown in Fig. 6.7 and the ratios shown in Fig. 6.8, with the ratios nearly always bounded by 0.96 and 1.04 and the relative standard uncertainties less than 4 %.

Comparing the results shown in Fig. 6.7 to those shown in Fig. 6.4(b), the convolution with a wider bandwidth obviously smoothes the spectral structure, as well as reduces the values of the maxima, of the relative standard uncertainty. However, some spectral structure corresponding to the spectral structure of the solar irradiance is still apparent, particularly at wavelengths between 315 nm and 320 nm. The relative standard uncertainty in the stability of the responsivity of the NSF instrument, obtained from the results shown in Fig. 5.9(d) in the same manner as for the Brewer instruments, is only 4 %. When this is combined with the relative standard uncertainties arising from random effects and from the responsivities of the other instruments, the combined relative standard uncertainty is indicating that the instrument responsivity was also increasing, which is consistent with the results shown in Figs. 5.9(d) and 5.10(d).

**Fig. 6.9:**
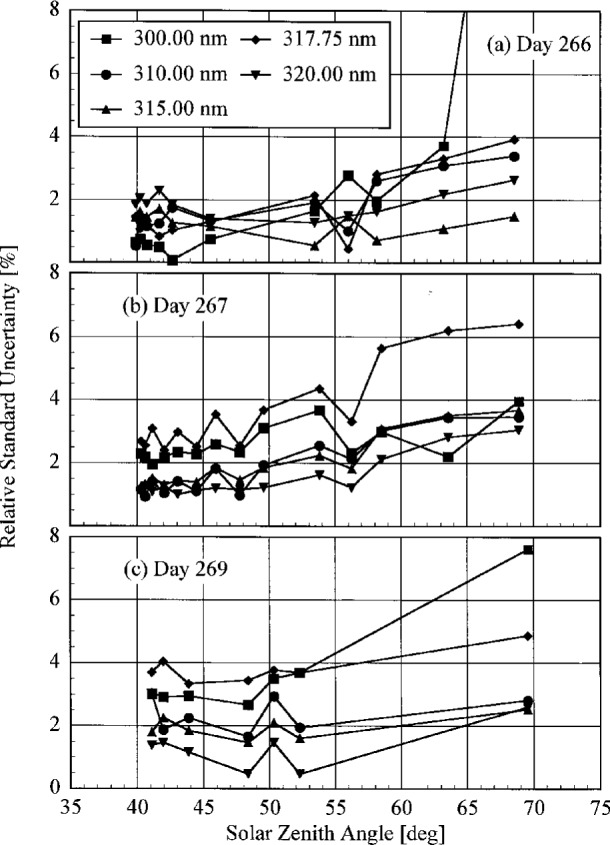
Relative standard uncertainty of the solar irradiances as a function of solar zenith angle at the wavelengths indicated in the legend determined by the four scanning instruments AES-1, AES-2, EPA, and NSF on the days indicated in the panels. The solar irradiances determined by each instrument were convolved with an ideal 1 nm FWHM triangular slit-scattering function.

The relative standard uncertainty of the solar irradiances as a function of solar zenith angle is shown in Fig. 6.9 on the days indicated in the panels at the wavelengths indicated in the legend. In contrast to the results shown in Fig. 6.6, there is a noticeable increase in the relative standard uncertainty with increasing solar zenith angle. This increase indicates a difference between the two types of instruments, probably in the Lambertian quality of their diffusers. Another possibility is a nonlinearity in one of the detectors. Without further information from instrument characterizations that were beyond the scope of the Intercomparison, the actual cause cannot be determined.

#### 6.4.4 All Instruments

The simplest approach for comparing the solar irradiances measured by all the instruments is to convolve the irradiances measured by the scanning instruments with the filter transmittances of the SERC instrument, all of which have approximately 2 nm bandwidths. This approach does not require any additional knowledge about the atmosphere, solar spectral irradiance, or radiative transfer. Therefore, the irradiance *E_j_* at filter channel *j* for each scanning instrument is given by

$$E_{j}=\sum_{i}E\left(\lambda \right)\tau_{j}\left(\lambda_{i}\right)/\sum_{i}\tau_{j}\left(\lambda_{i}\right),$$ (6.3)

where *i* indexes the wavelengths *λ_i_* and *τ_j_* is the filter transmittance for channel *j*. The relative standard uncertainty of the solar irradiances is calculated from the convolved irradiances from the four scanning instruments and from the measured irradiance by the SERC instrument.

**Fig. 6.10:**
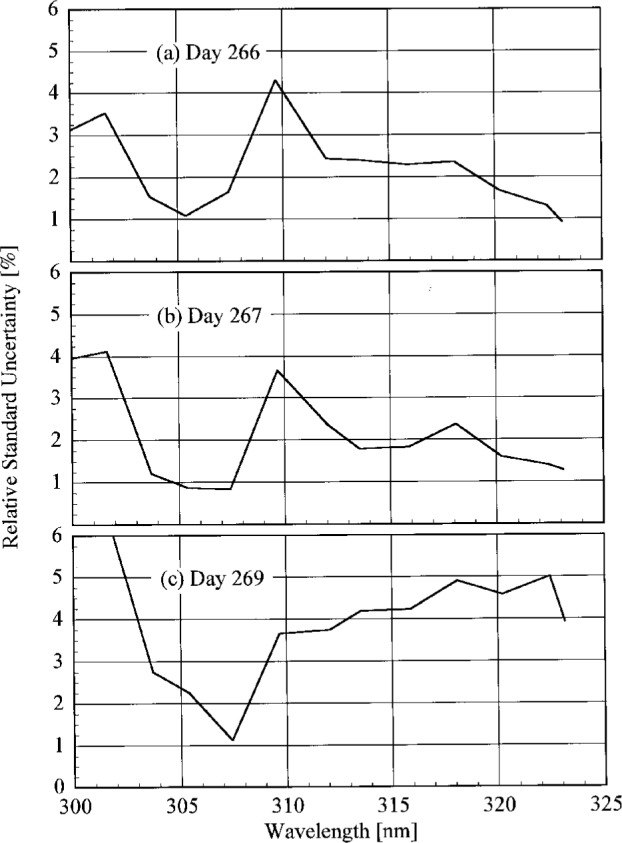
Relative standard uncertainty of the solar irradiances as a function of wavelength determined by all the instruments on the days indicated in the panels at 19:00 h. The solar irradiances determined by the scanning instruments were convolved with the transmittances of the filters of the SERC instrument.

**Fig. 6.11:**
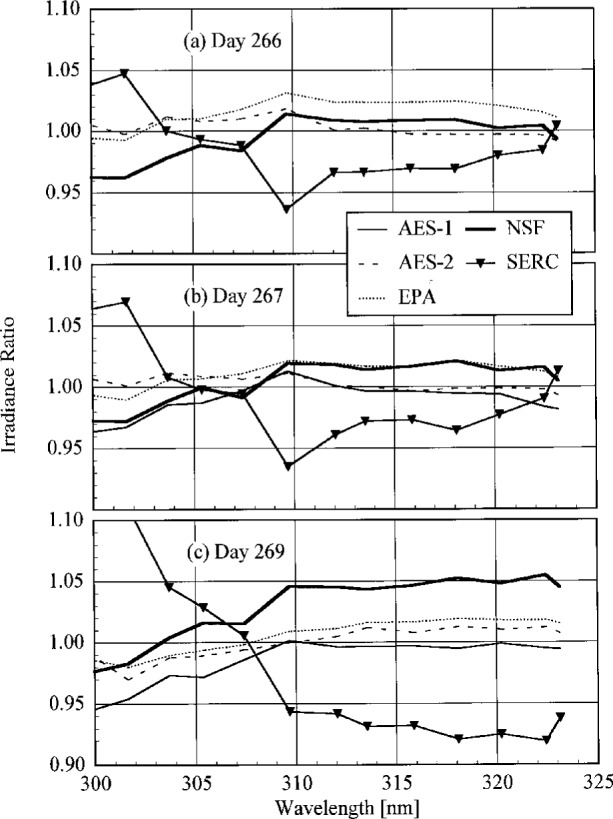
Ratio of solar irradiance measured by each instrument to the average irradiance as a function of wavelength by all the instrumentson the days indicated inthe panels at 19:00 h. The solar irradiances determined by the scanning instruments were convolved with the transmittances of the filters of the SERC instrument.

The relative standard uncertainty of the solar irradiances as a function of wavelength measured at 19:00 h is shown in Fig. 6.10 on the days indicated in the panels, and the corresponding irradiance ratios as a function of wavelength are shown in Fig. 6.11. The irradiances measured by the SERC instrument are consistently lower than those measured by the other instruments. As expected with such large bandwidths, there is very little spectral structure in the relative standard uncertainties. As with the results obtained with the scanning instruments, the relative standard uncertainties arising from random effects and changes in responsivity account for the values shown in Fig. 6.10.

**Fig. 6.12:**
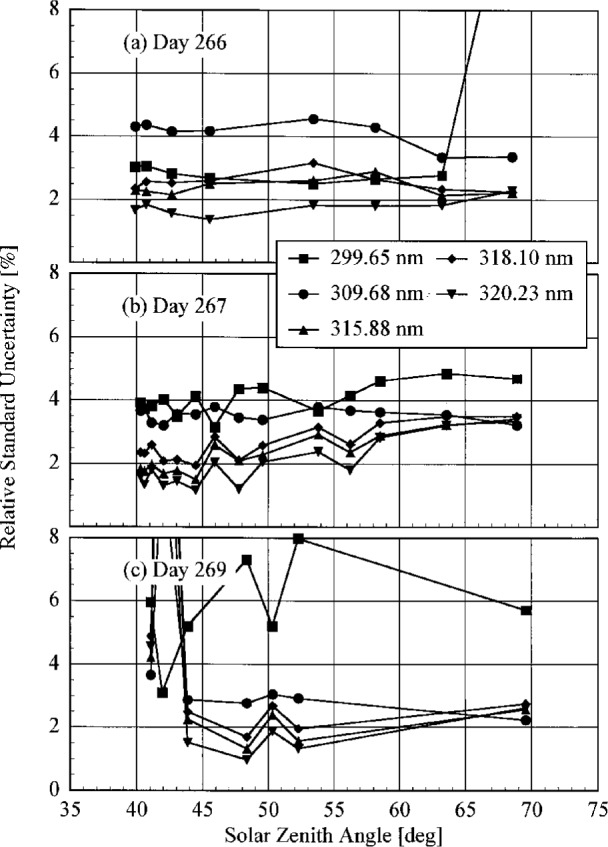
Relative standard uncertainty of the solar irradiances as a function of solar zenith angle at the wavelengths indicated in the legend determined by all the instruments on the days indicated in the panels. The solar irradiances determined by the scanning instruments were convolved with the transmittances of the filters of the SERC instrument.

The relative standard uncertainty of the solar irradiances as a function of solar zenith angle is shown in Fig. 6.12 on the days indicated in the panels at the wavelengths indicated in the legend. In contrast to the results shown in Fig. 6.9, there is no noticeable dependence of the relative standard uncertainties on solar zenith angle, which may be a result of the large bandwidths used in the convolution. Considering the simple approach used to compare all the instruments, the results are very encouraging. The relative standard uncertainties of the three Brewer instruments are generally 2 % or less, as shown in Figs. 6.6(a) and 6.6(b), except at 317.75 nm, where it is approximately 6 %. Expanding the comparison to include the NSF instrument increases the relative standard uncertainties to less than 4 %, as shown in Figs. 6.9(a), 6.9(b), and 6.9(c). Finally, including the SERC instrument in the comparison does not increase the relative standard uncertainties appreciably, as shown in Figs. 6.12(a), 6.12(b), and 6.12(c). In the best case, given by the results on day 267, the solar spectral irradiances agree to within 4 % for wavelengths from 300 nm to 320 nm.

#### 6.4.5 Comparison With Other Intercomparisons

The solar irradiances measured by the three Brewer instruments agree to within 6 % over the entire wavelength range of 300 nm to 325 nm, and the average agreement is 3 %. Including the NSF instrument and convolving with a 1 nm FWHM ideal triangular slit-scattering function brings the agreement between the measured irradiances to within 5 % even at large solar zenith angles. The agreement is not quite as good when the SERC instrument is included in the analysis.

The agreements between measured solar irradiances obtained for the North American Intercomparison represents a substantial improvement over the results from most other intercomparisons. The agreement between the solar irradiances at the second and third European intercomparison was within 10 % for most instruments, which was an improvement over the 20 % agreement at the first intercomparison [2–4]. The agreement obtained at the Finnish intercomparison was within 15 % [5], and within 10 % at intercomparisons held in New Zealand [6] and the Netherlands [7]. The results from the Netherlands intercomparison are interesting because the measured solar irradiance was deconvolved using the extraterrestrial irradiance and then convolved with a 1 nm FWHM ideal triangular slit-scattering function. While this is a more sophisticated technique for comparing the solar irradiances measured by instruments with different bandwidths than the technique used for the North American Intercomparison, the agreement between the irradiances is poorer. Most recently, the intercomparison in Germany [8] achieved agreement within 5 % using the spectral irradiance scales of the participants and convolution with a 5 nm FWHM ideal triangular slit-scattering function.

The most important advantage of the North American Intercomparison over the other intercomparisons was determining the responsivity of all the instruments outdoors on the pads with one standard lamp. Therefore, variations between spectral irradiance scales were removed from the measured solar irradiances, as well as changes in responsivity caused by moving the instruments or placing them on their sides. The intrinsic differences between instruments are thus primarily responsible for the variation of the measured solar irradiances.

## 7. Conclusions

The spectroradiometer characteristics that were easily assessed in the field and that are important for solar ultraviolet irradiance measurements were determined at the Intercomparison. Spectral scans of the emission lines from a Hg lamp and a HeCd laser were used for several purposes. Measurements of the slit-scattering functions of the scanning instruments showed that their bandwidths at 325 nm were close to their nominal values: 0.6 nm for the Brewer instruments and 0.95 nm for the NSF instrument. The bandwidths determined from the Hg lines generally decreased with increasing wavelength. The stray-light rejection, determined from scans of the HeCd laser, was at least 10^−4^ to 10^−5^ for the Brewer instruments, and 10^−5^ for the NSF and SERC instruments. These values for the NSF and SERC instruments were limited by the dynamic range of their signals. The wavelength accuracy of the scanning instruments was approximately 0.1 nm.

A standard lamp calibrated in the horizontal position with the NIST spectral irradiance scale was used to determine the responsivity of each instrument once indoors and twice outdoors. The irradiance scales of the participants agreed with the NIST scale to within 10 %, which is consistent with other Intercomparisons. The responsivities of the instruments changed upon moving them outdoors, probably from temperature differences for the Brewer instruments and mechanical shifts in the NSF instrument. The responsivities determined by the NIST standard lamp remained relatively constant outdoors, with the largest relative change being 4 % for the NSF instrument. The responsivities determined by the participants’ lamps were not as constant, probably due to temperature differences for the Brewer instruments. These results indicate the advantage of using a common irradiance standard for determining instrument responsivities and the necessity of performing these determinations at the locations where the instruments are measuring solar irradiance.

Synchronized solar irradiance scans from 290 nm to 325 nm were performed every half hour for several days of the Intercomparison. The results from days 266, 267, and 269 are useful as the weather was good and the instruments were operating properly. The relative standard uncertainties of the measured irradiances agreed well with the ratios of the irradiances to the average, and it was beneficial to summarize the relative standard uncertainties throughout a day as a function of solar zenith angle.

The solar irradiances measured by the three Brewer instruments agreed to within 6 % for wavelengths longer than 300 nm. The spectral structure of the relative standard uncertainties, which were consistent throughout a day, were correlated with the spectral structure of the solar irradiance. This indicated a wavelength uncertainty of 0.06 nm among the instruments, in addition to uncertainties arising from the signals and responsivities. The relative standard uncertainties were not dependent on the solar zenith angle, as expected for instruments with the same types of diffusers.

The solar irradiances measured by the four scanning instruments were compared by convolving all of them with a 1 nm FWHM ideal triangular slit-scattering function. The agreement between irradiances improved to within 4 % with the convolution, and the relative standard uncertainties increased slightly with solar zenith angle, indicating some difference in the angular responses of the diffusers. The SERC instrument was added to the comparison by convolving the irradiances measured by the scanning instruments with the filter transmittances of the SERC instrument. The agreement between irradiances increased slightly to within 5 %, and the relative standard uncertainties were independent of solar zenith angle.

This Intercomparison demonstrated that, when the spectral irradiance responsivities are determined consistently between instruments, it is possible to have the measured solar irradiances agree to within 5 % for wavelengths longer than 300 nm, even using a simple method for convolving the irradiances to a common bandwidth. This enables the intrinsic performance of the instruments to be assessed, and is an improvement over other Intercomparisons. Agreement of measured solar irradiances within 5 % seems to be a realistic goal for instruments in monitoring networks, given the uncertainties associated with the signals and responsivities, and changes in responsivity. All the participating instruments performed well, which is encouraging for future comparisons between instruments within the same ultraviolet monitoring network and between different networks.

## Figures and Tables

**Fig. 4.1(a):**
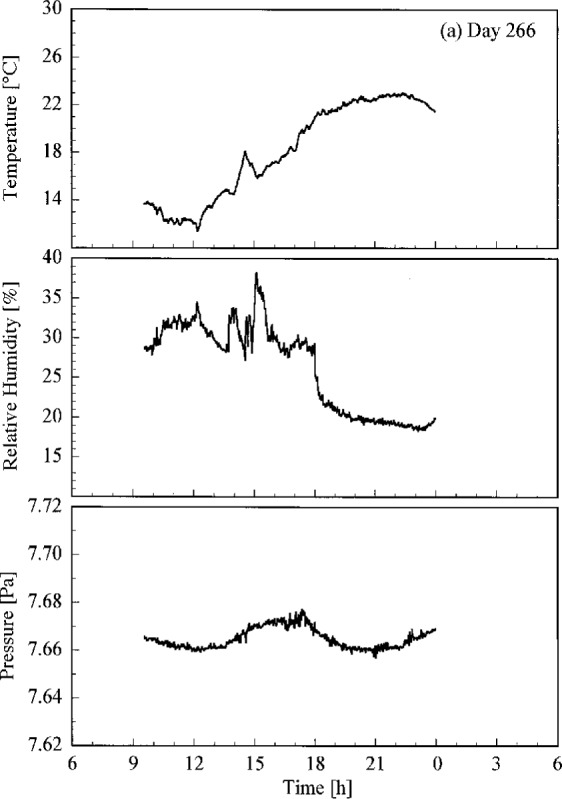
Temperature, relative humidity, and atmospheric pressure as a function of time on day 266 of the Intercomparison.

**Fig. 4.1(b):**
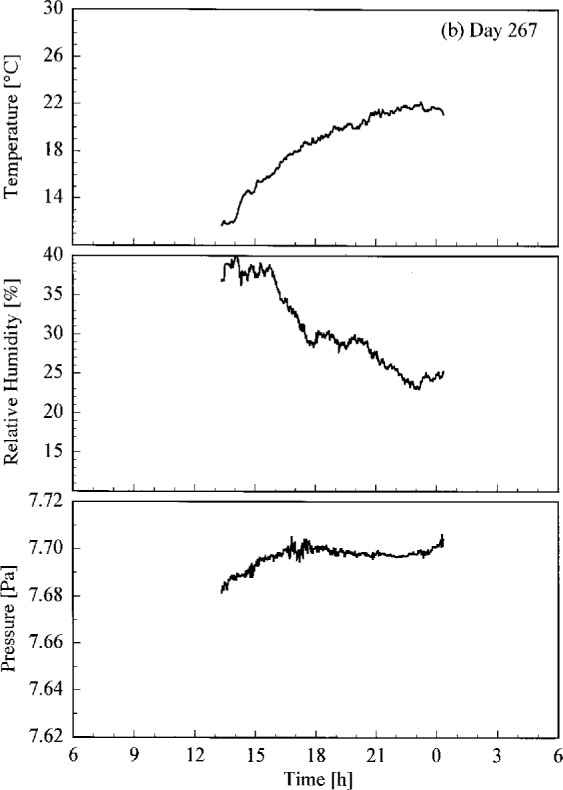
Temperature, relative humidity, and atmospheric pressure as a function of time on day 267 of the Intercomparison.

**Fig. 4.1(c):**
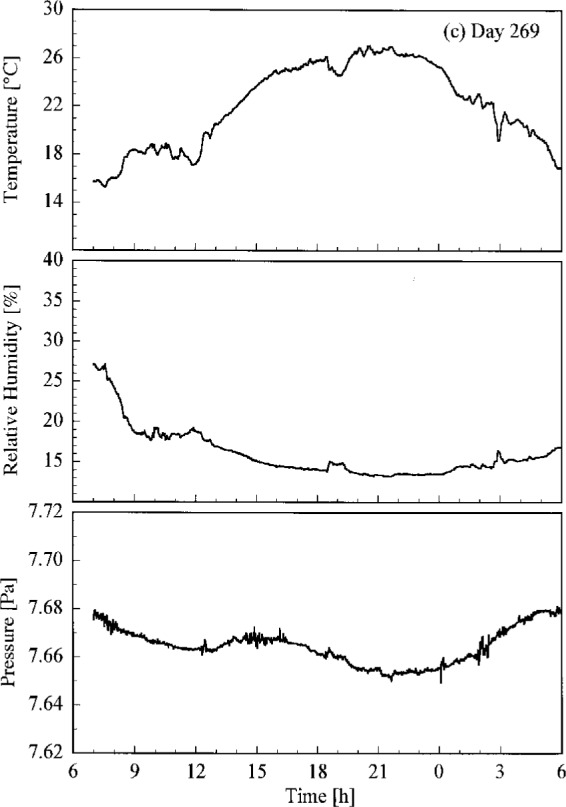
Temperature, relative humidity, and atmospheric pressure as a function of time on day 269 of the Intercomparison.

**Fig. 4.1(d):**
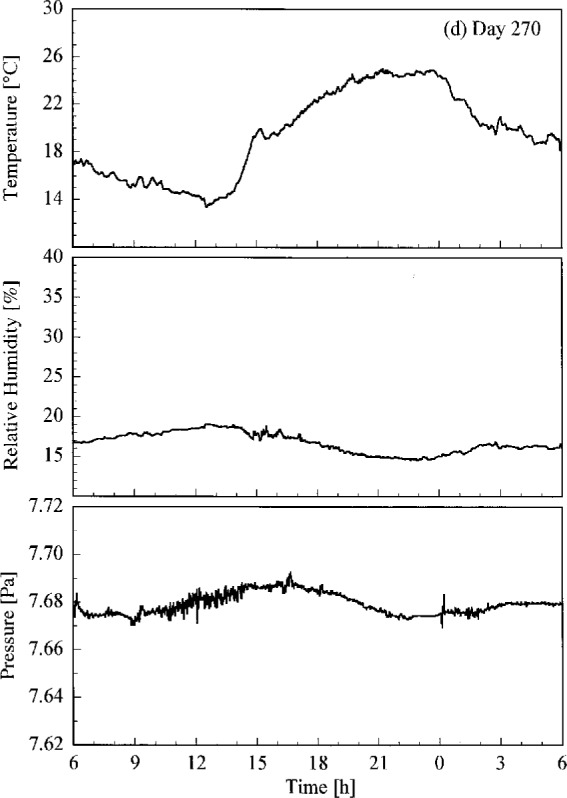
Temperature, relative humidity, and atmospheric pressure as a function of time on day 270 of the Intercomparison.

**Fig. 4.3(a):**
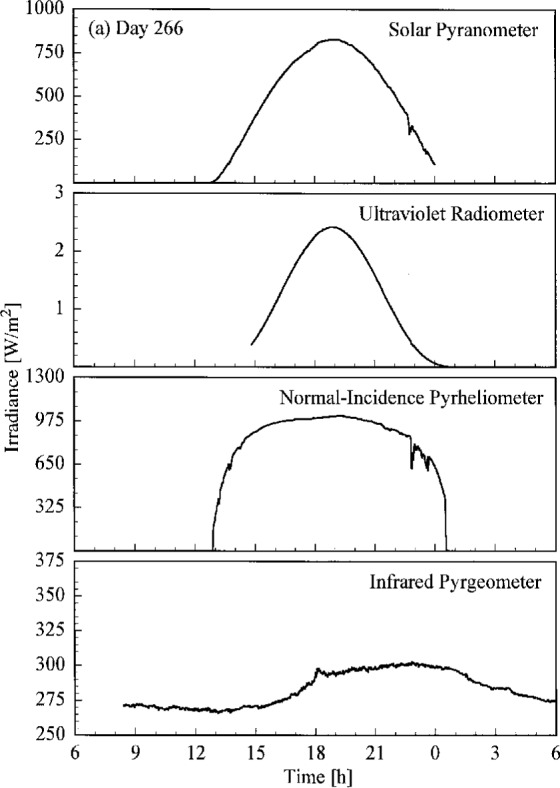
Irradiance as a function of time from the instruments indicated in the panels on day 266 of the Intercomparison.

**Fig. 4.3(b):**
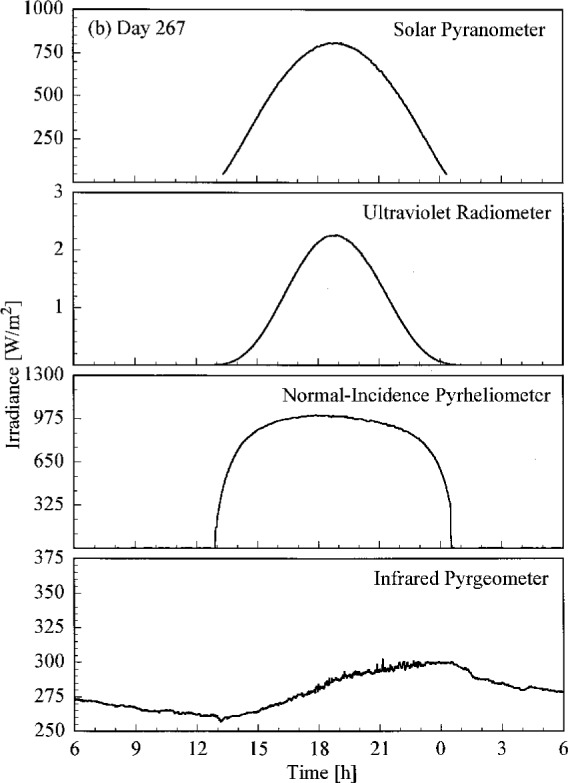
Irradiance as a function of time from the instruments indicated in the panels on day 267 of the Intercomparison.

**Fig. 4.3(c):**
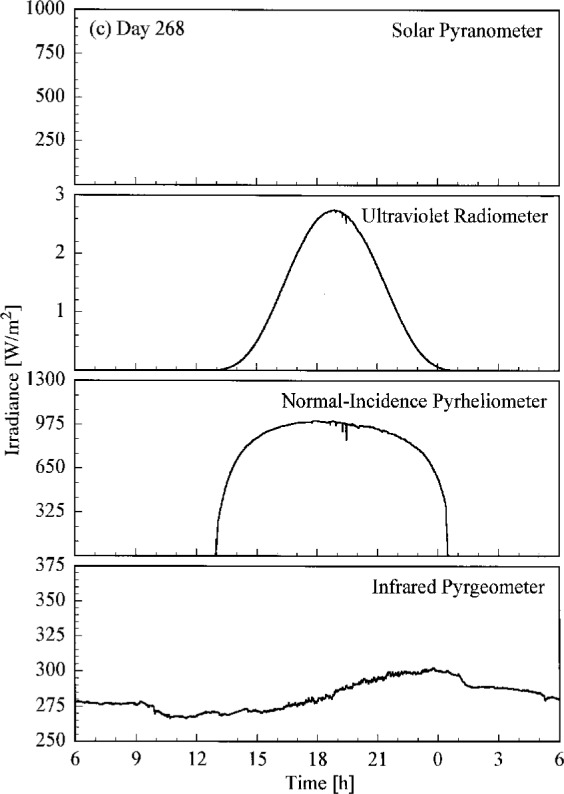
Irradiance as a function of time from the instruments indicated in the panels on day 269 of the Intercomparison.

**Fig. 4.3(d):**
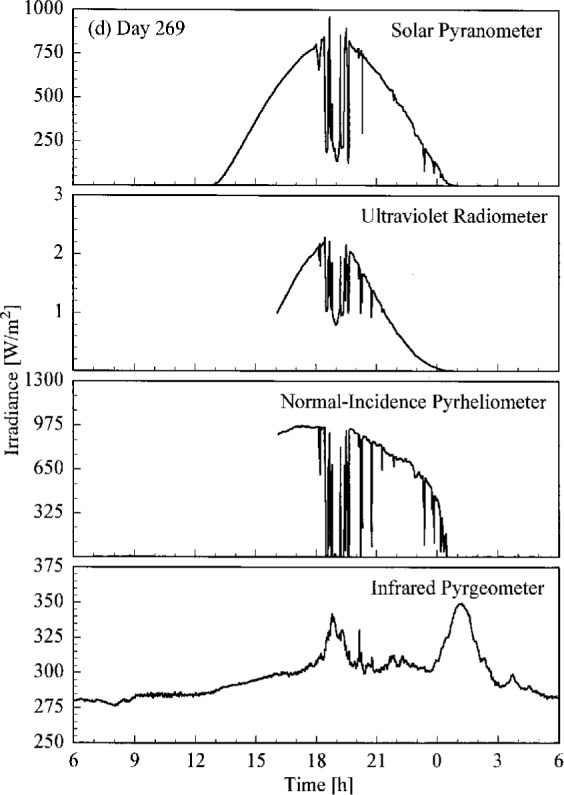
Irradiance as a function of time from the instruments indicated in the panels on day 270 of the Intercomparison.

**Fig. 4.3(e):**
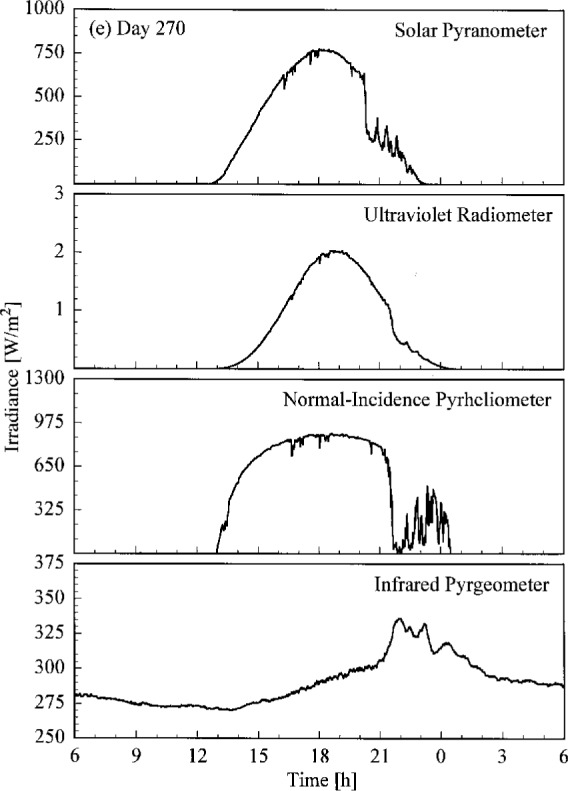
Irradiance as a function of time from the instruments indicated in the panels on day 270 of the Intercomparison.

**Table 1.1:** Instruments present during the 1994 North American Interagency Intercomparison of Ultraviolet Monitoring Spectroradiometers

Participating spectroradiometers		
Network	Instrument	Serial No.
AES	Sci-Tec Brewer MKII	009
AES	Sci-Tec Brewer MKII	113
EPA	Sci-Tec Brewer MKIV	109
NSF	BSI SUV-100	B-007
SERC	SERC SR-18	UC

Instrument	Ancillary instruments	Serial No.

Eppley Precision Solar Pyranmometer		73-38
Eppley Precision Solar Pyranmometer		73-44
Eppley Precision Solar Pyranmometer		73-99
Eppley Precision Infrared Pyrgeometer		29143
Eppley Precision Infrared Pyrgeometer		29144
Eppley Precision Infrared Pyrgeometer		29149
Eppley Normal Incidence Pyrheliometer		16665E6
Yankee UVB-1 Radiometer		940401
Yankee UVB-1 Radiometer		940402
Yankee UVB-1 Radiometer		940404
Solar Light Biometer		1501
Solar Light Biometer		1503
Solar Light Biometer		1506
Yankee Multi-Filter Rotating Shadowband		
Radiometer		8709
Yankee Ultraviolet Rotating Shadowband		
Radiometer		10150

Measurement	Meteorological instruments	Instrument

Temperature and relative humidity		Vaisala HMP 35C
Wind speed and direction		R. M. Young 05305
Barometric pressure		Vaisala PTB101B

**Table 3.1:** Spectroradiometer specifications

Participant	AES-1	AES-2	EPA	NSF	SERC
Spectroradiometer					
Model	Brewer	Brewer	Brewer	BSI	SERC
	MK II	MK II	MK IV	SUV-100	SR-18
Serial No.	009	113	109	B-007	UC
F/number	6	6	6	3.5	
Diffraction grating					
Number	1	1	1	2	
Type	plane	plane	plane	concave	
	holographic	holographic	holographic	holographic	
Lines per millimeter	1800	1800	1200	1200	
Blaze [nm]				250	
Diffraction order	second	second	third	first	
Dispersion	1 nm/mm	1 nm/mm	1 nm/mm	4 nm/mm	
PMT	9789QA	9789QA	9789QA	R-269	R-1657
Bandwidth [nm]	0.6	0.6	0.6	0.95	2 (nominal)
Step [nm]					
usual	0.5	0.5	0.5	0.2, 0.5, 1	2 (nominal)
finest	0.1	0.1	0.1	0.1	
Range [nm]	290 to 325	290 to 325	286 to 363	280 to 620	290 to 324
Diffuser material	Teflon	Teflon	Teflon	Teflon	Teflon
Weatherproof ?	Yes	Yes	Yes	Yes	Yes
Automatic ?	Yes	Yes	Yes	Yes	Yes
Temperature					
Stabilized optics ?	No	No	No	Yes	No
Stabilized detector ?	No	No	No	Yes	Yes
Dark current removed ?	Yes	Yes	Yes	Yes	Yes
Stray light removed ?	Yes	Yes	Yes	Yes	No
Wavelength regist. [nm]	302.3	302.3	302.3	296.7	
Primary lamp [W]	1000	1000	1000	200	1000
Secondary lamp [W]	50	50	50	45	

**Table 3.2:** Channel indicator, nominal and actual center wavelength, bandwidth, and maximum transmittance for each filter of SERC instrument UC

Channel	Nominal center wavelength (nm)	Actual center wavelength (nm)	Band-width (nm)	Maximum transmittance
A	290	289.93	2.25	0.077
B	292	291.92	2.45	0.063
C	294	293.70	2.40	0.086
D	296	295.62	2.25	0.077
E	298	297.38	2.35	0.065
F	300	299.65	2.30	0.065
G	302	301.65	2.30	0.084
H	304	303.70	2.70	0.101
I	306	305.40	2.10	0.110
J	Dark			
K	308	307.42	2.55	0.094
L	310	309.68	2.25	0.108
M	312	312.05	2.20	0.086
N	314	313.55	2.40	0.079
O	316	315.88	2.65	0.069
P	318	318.10	2.50	0.114
Q	320	320.23	2.65	0.104
R	322	322.45	2.40	0.115
S	324	323.15	2.40	0.107
T	Dark			

**Table 5.1:** Dates, lamps, times, and instrument temperatures of spectral scans determining responsivity

Instrument	Day	Lamp	Time (h)	Instrument temperature (°C)
AES-1	264	OS-27	15:00	29.6
		F-45	16:00	31.0
		U-4	17:00	32.2
		S-789	18:00	32.3
	267	S-789	1:00	27.1
	268	OS-27	4:45	14.1
		U-4	19:00	31.8
		S-789	19:45	32.3
		S-734	20:30	33.5
	270	U-4	16:45	25.6
		S-789	19:00	33.1
		S-789	21:30	37.4
		S-734	22:30	36.8
	271	OS-27	5:45	14.8
		U-4	7:00	16.7
AES-2	263	321	21:15	29.4
		322	21:45	30.7
		U-4	23:00	32.3
		OS-27	23:30	33.3
	264	F-45	0:30	33.8
	267	S-789	2:00	24.3
		321	23:30	32.5
	268	322	1:00	28.8
		OS-27	3:45	17.4
		321	5:00	16.3
		S-734	21:15	34.8
		S-789	22:00	35.1
		U-4	22:30	35.0
	271	S-789	3:45	20.4
		U-4	4:30	21.7
		OS-27	7:00	18.2
EPA	262	296	17:00	26.9
		296	17:30	28.4
		296	22:00	30.7
		OS-27	22:30	32.3
		GS-918	23:30	33.8
	263	F-45	0:30	35.0
		F-45	1:15	35.7
		296	20:45	33.8
	265	296	16:00	8.7
	268	OS-27	2:45	20.8
		296	3:45	19.3
	270	S-789	19:45	36.1
		S-789	20:15	36.8
		296	21:30	36.8
		296	21:45	36.4
		188	22:15	35.0
		188	22:45	34.6
		296	23:15	34.6
	271	298	2:45	21.2
		OS-27	3:30	32.3
		296	21:00	41.8
NSF	263	OS-27	15:15	32.5
		F-45	16:15	32.6
		M-761	17:30	32.6
		M-761	21:45	32.5
	265	M-761	22:15	32.5
	267	M-761	0:45	32.4
	268	M-761	1:00	32.3
		OS-27	6:00	32.4
		M-761	7:00	32.3
	270	OS-27	2:00	32.4
		M-761	3:00	32.3
SERC	267	OS-27	7:00	17.0
	271	OS-27	2:30	22.2

**Table 5.2:** Standard uncertainties from all components during responsivity measurements

Component	Wavelength [nm]	AES-1	Standard uncertainty		NSF	SERC
			AES-2	EPA		
Lamp						
Current		0.3 mA	0.3 mA	0.3 mA	0.3 mA	0.3 mA
Wavelength		0.1 nm	0.1 nm	0.1 nm	0.1 nm	0.1 nm
Alignment^a^						
Center						
diffuser		1.0 mm	1.0 mm	0.5 mm	0.5 mm	0.5 mm
jig		0.5 mm	0.5 mm	0.5 mm	0.5 mm	0.5 mm
Normal						
diffuser		1.5 mm	1.5 mm	1.0 mm	1.0 mm	1.0 mm
jig		2.0 mm	2.0 mm	2.0 mm	2.0 mm	2.0 mm
Distance		1.4 mm	1.4 mm	1.0 mm	1.0 mm	1.0 mm
Signal^b^,^c^	290	6.98 s^−1^	50.32 s^−1^	29.25 s^−1^	0.0110 nA	0.00160 V
	320	6.61 s^−1^	30.84 s^−1^	50.25 s^−1^	0.0113 nA	0.00028 V
	350			36.51 s^−1^	0.0065 nA	

a Standard uncertainties for aligning the diffuser and lamp jig normal to the optical axis are the maximum displacements of the retroreflected laser beam at distances of 110 cm and 60 cm, respectively.

b Standard uncertainties in the signal are from the scans used to calculate the responsivity used for solar irradiance measurements.

c The signals for AES-1, AES-2, and EPA instruments are photons per second.

**Table 5.3:** Relative standard uncertainties from all components during responsivity measurements

Component	Wavelength (nm)	AES-1	Relative standard uncertainty		NSF	SERC
			AES-2	EPA		
Lamp						
Irradiance	290	0.0117	0.0117	0.0117	0.0117	0.0117
	320	0.0102	0.0102	0.0102	0.0102	0.0102
	350	0.0090	0.0090	0.0090	0.0090	0.0090
Gonio.		0.0034	0.0034	0.0034	0.0034	0.0034
Current	290	0.0004	0.0004	0.0004	0.0004	0.0004
	320	0.0004	0.0004	0.0004	0.0004	0.0004
	350	0.0003	0.0003	0.0003	0.0003	0.0003
Wavelength	290	0.0038	0.0038	0.0038	0.0038	0.0038
	320	0.0031	0.0031	0.0031	0.0031	0.0031
	350	0.0024	0.0024	0.0024	0.0024	0.0024
Alignment						
Center		0.0006	0.0006	0.0004	0.0004	0.0004
Normal		0.0006	0.0006	0.0006	0.0006	0.0006
Distance		0.0029	0.0029	0.0016	0.0016	0.0016
Total		0.0030	0.0030	0.0018	0.0018	0.0018
Combined						
Lamp (*S*)^a^	290	0.0122	0.0122	0.0122	0.0119	0.0119
	320	0.0108	0.0108	0.0108	0.0105	0.0104
	350	0.0096	0.0096	0.0096	0.0093	0.0092
Lamp (*R*)^a^	290	0.0049	0.0049	0.0042	0.0042	0.0042
	320	0.0043	0.0043	0.0031	0.0031	0.0031
	350	0.0039	0.0039	0.0030	0.0030	0.0030
Signal (*R*)^a^	290	0.0007	0.0021	0.0050	0.0030	0.0020
	320	0.0005	0.0006	0.0044	0.0007	0.0032
	350			0.0059	0.0001	

a The components of relative standard uncertainty are obtained as follows: *R* designates a component arising from random effects, while *S* designates a component arising from systematic effects. The Lamp (*S*) values are the root-sum-square of the lamp irradiance and goniometric distribution relative standard uncertainties; the Lamp (*R*) values are the root-sum-square of the lamp current, instrument wavelength, and lamp alignment relative standard uncertainties; and the Signal (*R*) values are the relative standard uncertainties of the signals.

**Table 5.4:** Lamps, times, and temperature changes for responsivity ratios used for the figures

Figure	Numerator			Denominator			Temperature change (°C)
	Lamp	Day	Time (h)	Lamp	Day	Time (h)	
5.5(a)	OS-27	264	15:00	U-4	264	17:00	− 2.6
5.5(a)	OS-27	264	15:00	S-789	264	18:00	− 2.8
5.5(b)	OS-27	263	23:30	321	263	21:15	+3.9
5.5(b)	OS-27	263	23:30	322	263	21:45	+2.6
5.5(b)	OS-27	263	23:30	U-4	263	23:00	+0.9
5.5(c)	OS-27	262	22:30	296	262	22:00	+1.7
5.5(d)	OS-27	263	15:15	M-761	263	17:30	+0.1
5.6(a)	OS-27	271	5:45	U-4	271	7:00	− 1.9
5.6(b)	OS-27	268	3:45	321	268	5:00	+1.1
5.6(b)	OS-27	271	7:00	S-789	271	3:45	− 2.2
5.6(b)	OS-27	271	7:00	U-4	271	3:30	− 3.5
5.6(c)	OS-27	268	2:45	296	268	3:45	+ 1.5
5.6(d)	OS-27	268	6:00	M-761	268	7:00	+ 0.1
5.7(a)	OS-27	268	4:45	OS-27	264	15:00	− 15.5
5.7(b)	OS-27	268	3:45	OS-27	263	23:30	− 15.8
5.7(c)	OS-27	268	2:45	OS-27	262	22:30	− 11.6
5.7(d)	OS-27	268	6:00	OS-27	263	15:15	− 0.1
5.8(a)	S-789	267	1:00	S-789	264	18:00	− 5.2
5.8(b)	321	267	23:30	321	263	21:15	+ 3.2
5.8(b)	322	268	1:00	322	263	21:45	− 1.9
5.8(c)	296	265	16:00	296	262	22:00	− 22.0
5.8(d)	M-761	265	22:15	M-761	263	17:30	− 0.1
5.9(a)	OS-27	271	5:45	OS-27	268	4:45	+ 0.7
5.9(b)	OS-27	271	7:00	OS-27	268	3:45	+ 0.8
5.9(c)	OS-27	271	3:30	OS-27	268	2:45	+11.6
5.9(d)	OS-27	270	2:00	OS-27	268	6:00	0
5.9(e)	OS-27	271	2:30	OS-27	267	6:00	+ 5.3
5.10(a)	S-789	268	19:45	S-789	267	1:00	+5.2
5.10(a)	S-789	170	19:00	S-789	268	19:45	+0.8
5.10(a)	S-789	270	21:30	S-789	170	19:00	+4.3
5.10(a)	U-4	270	16:45	U-4	268	19:00	− 6.2
5.10(a)	U-4	271	7:00	U-4	270	16:45	− 8.9
5.10(a)	S-734	270	22:30	S-734	268	20:30	+ 3.4
5.10(b)	S-789	268	22:00	S-789	267	2:00	+ 10.8
5.10(b)	S-789	271	3:45	S-789	268	22:00	− 14.7
5.10(b)	U-4	271	4:30	U-4	268	22:30	− 13.2
5.10(b)	321	268	5:00	321	267	23:30	− 16.2
5.10(c)	296	268	3:45	296	265	16:00	+ 10.6
5.10(c)	296	270	21:45	296	268	3:45	+ 17.2
5.10(c)	296	271	21:00	296	270	21:45	+ 5.4
5.10(d)	M-761	270	3:00	M-761	268	7:00	0

**Table 6.1:** Days, times, and participating instruments of synchronized spectral scans of solar ultraviolet irradiance. Days 266, 267, and 269 are Sept. 23, 24, and 26, 1994, respectively

Day	Time (h)	AES-1	Participating instruments		NSF	SERC
			AES-2	EPA		
266	16:00		X	X	X	
	18:00		X	X	X	
	18:30		X	X	X	
	19:00		X	X	X	X
	19:30		X	X	X	X
	20:00		X	X	X	X
	20:30		X	X	X	X
	21:30		X	X	X	X
	22:00		X	X	X	X
	22:30		X	X	X	X
	23:00		X	X	X	X
267	16:00	X	X	X	X	X
	17:00	X	X	X	X	X
	17:30	X	X	X	X	X
	18:00	X	X	X	X	X
	18:30	X	X	X	X	X
	19:00	X	X	X	X	X
	19:30	X	X	X	X	X
	20:00	X	X	X	X	X
	20:30	X	X	X	X	X
	21:00	X	X	X	X	X
	21:30	X	X	X	X	X
	22:00	X		X	X	X
	22:30	X		X	X	X
	23:00	X		X	X	X
	23:30			X	X	X
269	16:30	X	X	X	X	X
	17:00	X	X	X	X	X
	19:00	X	X	X	X	X
	19:30	X	X	X	X	X
	20:00	X	X	X	X	X
	21:00	X	X	X	X	X
	21:30	X	X	X		X
	22:00	X	X	X		X
	23:00	X	X	X	X	X

## Footnotes

1 Certain commercial equipment, instruments, or materials are identified in this paper to foster understanding. Such identification does not imply recommendation or endorsement by the National Institute of Standards and Technology, nor does it imply that the materials or equiment identified are necessarily the best available for the purpose.

## 8. Appendix A. Attendees

The following people attended the 1994 North American Interagency Intercomparison of Ultraviolet Monitoring Spectroradiometers, and are grouped by function and network.

**Table:**

Coordinators		
National Institute of Standards and Technology (NIST)		
NIST		
Bldg. 220, Rm. A-320		
Gaithersburg, MD 20899-0001		
Ambler Thompson	(301) 975-2333	ambler@enh.nist.gov
Ted Early	(301) 975-2343	early@enh.nist.gov
Fax	(301) 840-8551	
National Oceanic and Atmospheric Administration (NOAA)		
NOAA R/E/ARx1		
325 Broadway		
Boulder, CO 80303		
John DeLuisi	(303) 497-6083	deluisi@srrb.noaa.gov
Patrick Disterhoft	(303) 497-6355	dister@srrb.noaa.gov
Fax	(303) 497-6546	
Participants		
Atmospheric Environment Service (AES)		
Environment Canada		
4905 Dufferin Street		
Toronto, ON M3H 5T4 Canada		
David Wardle	(416) 739-4632	dwardle@dow.on.doe.ca
Jim Kerr	(416) 739-4626	jkerr@dow.on.doe.ca
Fax	(416) 739-4281	
Environmental Protection Agency (EPA)		
University of Georgia		
Dept. of Physics and Astronomy		
University of Georgia		
Athens, GA 30602		
John Rives	(706) 542-5755	jrives@hal.physast.uga.edu
Yongchen Sun	(706) 542-1396	ysun@uga.cc.uga.edu
Fax	(706) 542-2492	
National Science Foundation (NSF)		
Biospherical Instruments, Inc.		
5340 Riley Street		
San Diego, CA 92110-2621		
Timothy Lucas	(619) 686-1888	lucas@biospherical.com
Michael Duhig	(619) 686-1888	duhig@biospherical.com
Fax	(619) 686-1887	
Smithsonian Environmental Research Center (SERC)		
Smithsonian Environmental Research Center		
P.O. Box 28		
Edgewater, MD 21037		
Patrick Neale	(410) 798-4424	neale@serc.si.edu
Fax	(301) 261-7954	
Observers		
United States Department of Agriculture (USDA)		
Atmospheric Sciences Research Center		
University at Albany		
State University of New York		
100 Fuller Road		
Albany, NY 12205		
Lee Harrison	(518) 442-3811	lee@solsun1.asrc.albany.edu
Jerry Berndt	(518) 442-3788	jerry@solsun1.asrc.albany.edu
Fax	(518) 442-3867	
Johns Hopkins Applied Physics Lab (APL)		
The Johns Hopkins University		
Applied Physics Laboratory		
Johns Hopkins Road		
Laurel, MD 20723-6099		
Steven Lloyd	(301) 953-5000, x8164	steven_lloyd@jhuapl.edu
Sam Yee	(301) 953-5000	jeng-hwa_yee@jhuapl.edu
Fax	(301) 953-6670	
Aerological Observatory		
Aerological Observatory,		
J.M.A. 2-1 Nagamine,		
Tsukuba, Ibaraki 305 Japan		
Masanori Shitamichi	81-0298-51-4127	
Fax	81-0298-51-5765	

## 9. Appendix B. Upper-Air Conditions

On the morning of Sept. 20 (day 263) at 12:00 h upper troposheric conditions were rather benign. The central United States was under the influence of a broad ridge flanked by two weak upper-level troughs over the southwest and southeast United States. Winds at 30 kPa were uncharacteristically light over the entire country. However, at the same time, an energetic undulating pattern was governing the weather over Canada. Of specific interest was a sharp upper-level shortwave trough over Alberta. A northerly confluent jet streak greater than 46 m/s was west of the trough line over British Columbia. At 50 kPa there was strong positive vorticity advection in the northerly flow on the back side of the trough. The surface low associated with this wave was in northern Saskatchewan. The position of the surface low to the east of the upper-level trough in Alberta indicated that the storm had a westward tilt with height. This, together with strong mid-tropospheric vorticity advection, signaled rapid intensification of the storm.

Within 12 h, the amplitude of the upper-level wave increased and the maximum 30 kPa winds on the back side of the trough increased to greater than 62 m/s. During the next 12 h the upper-level wave “dug” southeastward through Wyoming and Colorado, and by 12:00 h on Sept. 23 (day 266) it had “cut off” over eastern Nebraska, leaving the westerlies well to the north in Canada. On the morning of Sept. 22 (day 265), the mid-and upper-level low was stacked vertically over eastern Nebraska and the surface low was a few hundred km to the east over Iowa. By that time the jet stream at 30 kPa had wrapped completely around the low center and vorticity advection at 50 kPa had become negligible. These conditions pointed to a quasi-stationary weather pattern over the United States for several days to come. Accordingly, through Sept. 22 (day 265) and early on the morning of Sept. 23 (day 266), the surface low occluded and drifted slowly in a counter clockwise path from Iowa to southern Missouri. By that time the storm system was vertically stacked through the depth of the troposphere. Over the next few days it remained that way and far removed from the westerlies, which were several hundred km to the north in Canada. With no push from the west and no propagation mechanism (i.e., no vorticity advection), the storm spun virtually in place over the next several days.

On the morning of Sept. 24 (day 267), the 50 kPa low was over southern Missouri, directly over the surface low position. Northerlies on the west side of this large storm extended through Kansas, Colorado, and Utah. The quasi-geostrophic forcing for vertical motion (thermal advection plus differential vorticity advection) brought downward motion through Oklahoma, Kansas, Nebraska, and eastern Colorado. Regions of upward motion forcing were mainly north, east, and southeast of the low center, and are associated with clouds. As these clouds translated around the low, satellite imagery showed them dissipate as they entered this large region of downward forcing.

By the next day, Sept. 25 (day 268), the middle-level low gradually began to open and move northeastward but, owing to the storm’s slow movement and north-northeastward track, northeastern Colorado remained in deep northerly flow for the next several days. On the last day of the Intercomparison, the upper-level trough was over the Great Lakes, but an upper-level ridge continued to dominate the High Plains and the intermountain west. It was not until Sept. 30 that an upper-level trough finally moved in from the west to replace the longstanding ridge that had dominated northeastern Colorado’s weather for more than a week.

## References

[1] (1995). The U.S. Interagency UV-Monitoring Network Plan. United States Global Change Research Program.

[2] Gardiner BG, Webb AR, Bais AF, Blumthaler M, Dirmhirn I, Forster P, Gillotay D, Henridsen K, Huber M, Kirsch PJ, Simon PC, Svenoe T, Weihs P, Zerefos CS (1993). European Intercomparison of Ultraviolet Spectroradiometers. Env Tech.

[3] Gardiner BG, Kirsch PJ (1994). Second European Intercomparison of Ultraviolet Spectroradiometers: Report to the Commission of the European Communities.

[4] Gardiner BG, Kirsch PJ (1995). Setting Standards for European Ultraviolet Spectroradiometers: Report to the Commission of the European Communities.

[5] Koskela T (1994). The Nordic Intercomparison of Ultraviolet and Total Ozone Instruments at Izaña from 24 October to 5 November 1993.

[6] McKenzie RL, Kotkamp M, Seckmeyer G, Erb R, Roy CR, Gies HP, Toomey SJ (1993). First Southern Hemisphere Intercomparison of Measured Solar UV Spectra. Geophys Res Lett.

[7] Slaper H, Reinen HAJM, Blumthaler M, Huber M, Kuik F (1995). Comparing Ground-Level Spectrally Resolved Solar UV Measurements Using Various Instruments: A Technique Resolving Effects of Wavelength Shift and Slit Width. Geophys Res Lett.

[8] Seckmeyer G, Mayer B, Bernhard G, McKenzie RL, Johnston PV, Kotkamp M, Booth CR, Lucas T, Mestechkina T, Roy CR, Gies HP, Tomlinson D (1995). Geographical Differences in the UV Measured by Intercompared Spectroradiometers. Geophys Res Lett.

[9] Booth CR, Lucas TB, Mestechkina T, Tusson JR, Neuschuler DA, Morrow JH (1993). NSF Polar Programs UV Spectroradiometer Network 1991–1993 Operations Report.

[10] Booth CR, Lucas TB, Morrow JH, Weiler CS, Penhale PA (1994). The United States National Science Foundation’s Polar Network for Monitoring Ultraviolet Radiation. Antarctic Res Ser.

[11] Correll DL, Clark CO, Goldberg B, Goodrich VR, Hayes DR, Klein WH, Schecher WD (1992). Spectral Ultraviolet-B Radiation Fluxes at the Earth’s Surface: Long-Term Variations at 39 °N, 77 °W. J Geophys Res.

[12] Sansonetti CJ, Salit ML, Reader J (1996). Wavelengths of Spectral Lines in Mercury Pencil Lamps. Appl Optics.

[13] Reader J, Sansonetti CJ, Bridges JM (1996). Irradiances of Spectral Lines in Mercury Pencil Lamps. Appl Optics.

[14] Early EA, Thompson EA (1996). Irradiance of Horizontal Quartz-Halogen Standard Lamps. J Res Natl Inst Stand Technol.

[15] Walker JH, Thompson EA (1994). Improved Automated Current Control for Standard Lamps. J Res Natl Inst Stand Technol.

[b16-j23tho] 16Neale, private communication.

